# Structural Variation-Associated Expression Changes Are Paralleled by Chromatin Architecture Modifications

**DOI:** 10.1371/journal.pone.0079973

**Published:** 2013-11-12

**Authors:** Nele Gheldof, Robert M. Witwicki, Eugenia Migliavacca, Marion Leleu, Gérard Didelot, Louise Harewood, Jacques Rougemont, Alexandre Reymond

**Affiliations:** 1 Center for Integrative Genomics, University of Lausanne, Lausanne, Switzerland; 2 Swiss Institute of Bioinformatics (SIB), Lausanne, Switzerland; 3 School of Life Sciences, Ecole Polytechnique Fédérale de Lausanne, Lausanne, Switzerland; Florida State University, United States of America

## Abstract

Copy number variants (CNVs) influence the expression of genes that map not only within the rearrangement, but also to its flanks. To assess the possible mechanism(s) underlying this “neighboring effect”, we compared intrachromosomal interactions and histone modifications in cell lines of patients affected by genomic disorders and control individuals. Using chromosome conformation capture (4C-seq), we observed that a set of genes flanking the Williams-Beuren Syndrome critical region (WBSCR) were often looping together. The newly identified interacting genes include *AUTS2*, mutations of which are associated with autism and intellectual disabilities. Deletion of the WBSCR disrupts the expression of this group of flanking genes, as well as long-range interactions between them and the rearranged interval. We also pinpointed concomitant changes in histone modifications between samples.

We conclude that large genomic rearrangements can lead to chromatin conformation changes that extend far away from the structural variant, thereby possibly modulating expression globally and modifying the phenotype.

GEO Series accession number: GSE33784, GSE33867.

## Introduction

Copy number variation (CNV) of genomic segments is frequent in human [1] and model organisms (e.g. mouse [2]–[6]). More than 66,000 human CNVs mapping to 16,000 regions have so far been identified (http://projects.tcag.ca/variation/). They significantly contribute to genetic variation, covering more nucleotide content per genome than single nucleotide polymorphisms (e.g. approximately 0.8% of the length of the human genome differs between two human individuals [7]). Multiple associations between these structural changes and susceptibility to disease have been uncovered (reviewed in [8]–[12]). One of these is the Williams-Beuren syndrome, a multi-system disorder caused by a recurrent megabase-scale segmental deletion (WBS, MIM ID #194050, [13]).

CNVs impact tissue transcriptomes on a global scale by modifying the level and timing of expression of genes that localize within the CNV [14], [15] and on its flanks [5], [6], [16]–[18], an effect that can extend over the entire length of the affected chromosome [19]. Structural changes *per se*, i.e. without changes in gene dosage were shown to profoundly impact the phenotypic outcome, as some phenotypic traits present in Smith-Magenis (deletion) and Potocki-Lupski syndromes (reciprocal duplication) mouse models were not rescued by restoration of the copy number in a strain carrying both the deletion and duplication on different alleles [19]. The mechanism(s) behind this chromosome-wide effect is(are) currently unknown. One hypothesis is that some of the genes that map within a rearrangement, and thus vary in dosage, directly or indirectly affect the expression of normal dosage flanking genes. However, as in multiple instances we found the flanking genes to be altered independent of CNV dosage (i.e. both the deletion of a given CNV and its reciprocal duplication upregulate the expression of a flanking gene)[19], [20], it is unlikely that this hypothesis constitute the only mechanism behind this “neighboring effect”. Other mechanisms may include position effect (i.e. physical dissociation of a transcription unit from its *cis*-acting regulators [21]), alteration of chromatin structure locally or globally [22], and/or repositioning of a genomic region within the nucleus [23].

As chromatin structure plays an important role in gene regulation, we anticipate that CNVs will affect the chromatin structure on a large scale, and hence possibly modify the clinical phenotype. However, studies investigating the impact of a structural aberration on long-range chromatin structure have been lacking. Here, we explored the chromosome-wide effect of a structural rearrangement on chromatin structure. First, we studied, by chromosome conformation capture, whether non-hemizygous genes neighboring a rearrangement and known to be affected in their expression levels also show changes in chromatin structure. Second, we monitored local chromatin changes as determined by histone modifications in the same cell lines with a structural rearrangement.

## Results

### Outlining the chromatin architecture of the WBS region

**Figure 1:**
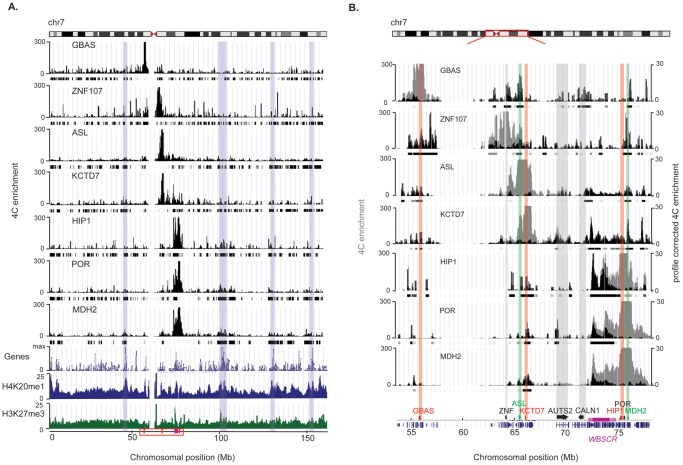
Extensive chromatin interactions of seven genes flanking the WBSCR on human chromosome 7 (HSA7) in cells from a healthy control individual. (**A**) Windowed and normalized 4C signal of each of the seven viewpoints along the entire HSA7. The black ticks below each graph show the location of the Bricks (Blocks of Regulators In Chromosomal Kontext). The gene density across HSA7, as well as the windowed profiles of H4K20me1 and H3K27me3 marks in the same cell line are shown below. Some examples of strong correlation of gene-dense regions and high density of H4K20me1 marks with highly interacting regions are highlighted in blue. The mapping of the assessed genes/viewpoints and of the WBSCR is indicated at the bottom. The red box specifies the close-up shown in panel B. (**B**) Close-up of the windowed 4C signal of the seven viewpoints around the WBSCR for the region indicated with a red box on HSA7 (top panel). The windowed 4C signal is shown in grey, while the profile corrected 4C signal (after removal of the highly interacting neighboring background signal) is overlaid in black. The position of all genes are displayed at the bottom, and the mapping of the assessed viewpoints is highlighted by red and green arrows indicating if the corresponding genes are down- or upregulated in cells from WBS patients, respectively. Black arrows underscore the mapping of the viewpoint that is not modified in gene expression (*ZNF107*) and the newly identified interacting partners *AUTS2* and *CALN1*. The location of the WBSCR is indicated by a purple horizontal bar. A close-up of interactions within this WBSCR is provided in **Supplementary Figure S4**.

We have previously shown that *GBAS, ASL, KCTD7, HIP1, POR* and *MDH2* (normal-copy number genes that map to the flank of the 7q11.23 deletion that causes WBS) are modified in their relative expression levels in lymphoblastoid and/or skin fibroblast cell lines of WBS patients [16]. We replicated these experiments in a new set of lymphoblastoid cell lines (**Table 1**). To assess if these changes are associated with changes in chromatin conformation, we examined the chromatin interaction landscape of these six flanking genes in the same lymphoblastoid cells using an adaptation of the 4C method (4C-seq: Circularized Chromosome Conformation Capture combined with multiplexed high-throughput sequencing). This technology allows identification of chromosomal regions that physically associate with a given locus, termed the bait or viewpoint. We included an additional viewpoint at the transcriptional start site of *ZNF*107, a gene located between the *GBAS* and *ASL* that did not show any significant change in expression in WBS versus Control cell lines (**Table 1**) [16]. **Figure 1A** shows the windowed interaction profiles for each viewpoint along the entire human chromosome 7 (HSA7) in the Ctrl cell line. Results are highly reproducible (0.83≤ Pearson's correlation ≤0.97; **Supplementary Figure S1**). After removal of the strong local background signal, we used a statistical segmentation algorithm to detect significantly interacting regions without imposing a fixed window size (see methods) [24], [25]. A stringent and a relaxed false discovery rate were imposed to detect “long-” and “short-range” interactions (within a 25 Mb region encompassing the WBS deletion), respectively. We identified between 66 and 152 interacting regions on HSA7 for the seven tested viewpoints (**Supplementary Table S2**).

**Table 1:** Expression changes and chromatin architecture modifications in WBS cells.

		Expression (this work)						Expression (ref 16)		H4K20me1 changes *	H3K27me3 changes
Gene	Category	Ctrl		WBS		WBS/Ctrl		WBS/Ctrl			
		AREL	SD	AREL	SD	AREL ratio	*t* Test *P*	AREL ratio	*t* Test *P*		
GBAS	viewpoint	0.874	0.095	0.431	0.010	0.493	0.014	0.74	0.02	−0.67	NS
ASL	viewpoint	0.033	0.005	0.046	0.004	1.424	0.029	1.59	0.004	1	NS
KCTD7	viewpoint	0.067	0.006	0.061	0.003	*0.922*	*0.272*	0.39	0.004	*−0.1*	NS
HIP1	viewpoint	0.119	0.019	0.048	0.013	0.403	0.009	0.47	0.02	0.81	1.38
POR	viewpoint	0.241	0.064	0.201	0.029	*0.833*	*0.401*	*0.89*	*0.37*	−0.73	NS
MDH2	viewpoint	9.229	0.321	12.32	0.195	1.335	0.0004	1.23	0.002	*−0.22*	NS
ZNF107	control viewpoint	20.90	2.717	21.87	1.701	*1.046*	*0.633*	*0.85*	*0.24*	−0.96	NS
AUTS2	novel interactor	2.739	0.101	0.680	0.006	0.248	0.001	0.35	0.06	−1.55	2.58
CALN1	novel interactor	BDL		BDL				BDL		−0.45	0.77
WBSCR22	positive control	0.277	0.031	0.125	0.024	0.451	0.003	0.43	0.0003	−1.67	NS

Changes in expression and chromatin structure in WBS (GM13472) versus Ctrl (GM07006) cells. Changes in histone marks are presented as the log2-fold ratio between WBS and Ctrl cells. Statistical analysis was performed by a 2-sample t-Test. Values in italics are not statistically different.

AREL  =  average relative expression level, BDL  =  below detection line, NS  =  no regions within gene were defined as significantly changed,

*most significant block according to SICER within the gene (FDR<1%).

We assessed whether our data are consistent with known features of chromosome conformation. As previously published we observed the strongest interactions close to the viewpoints, a clustering of gene-dense regions and possible regulatory regions and that loci interact more frequently with regions along the same chromosomal arm [26], [27] (**Figure 1A**). Chromosome-wide interactions of all viewpoints are significantly enriched in gene-dense regions (*P* = 0.09 for *GBAS*, *P*<0.05 for all other 6 viewpoints, permutation test with number of permutations N = 10000). We also found a positive correlation between the number of viewpoints with which a region interacts and the gene density of that particular region: regions interacting with all, five (excluding *GBAS*), two or only a single viewpoint(s) have a gene density of 4.8×10^−2^, 4.1×10^−2^, 1.7×10^−2^, 0.3×10^−2^ RefSeq genes/kilobase, respectively. We then compared chromosome-wide interactions with the ENCODE data set of expressed genes from the GM12878 lymphoblastoid cell line [28], [29] and found significant enrichment in expressed genes (*P* = 0.25 for *ZNF107*, and *P*<0.05 for all other viewpoints, permutation test with number of permutations N = 1000). We also investigated whether regulatory elements were enriched in the interacting regions. Towards this goal we used the ENCODE datasets of different regulatory marks from the same GM12878 cell line including H3K4me1, H3K27ac, p300, CTCF, DNaseI hypersensitive sites (DHSs) and FAIRE sites [29], [30]. Not only the expressed genes, but also these marks of functional elements were significantly enriched in the interacting regions at all viewpoints except for p300 (**Supplementary Table S3**). A large fraction of the interacting regions are shared between multiple viewpoints on the long arm of HSA7 (**Supplementary Figure S3**). For example, 23% (28/121) of the regions found to interact with *POR* also interact with the *ASL, KCTD7*, *HIP1* and *MDH2* viewpoints. They cluster however less with the *GBAS* viewpoint, which maps to the short arm of HSA7. An exception is *ZNF107*, which maps close to the centromere, and interacts frequently with the other side of the centromere. The robustness of the 4C assays is finally further exemplified by the fact that many of the reported interactions are identified reciprocally (see below).

We next zoomed in on the interaction profiles of the viewpoints around the WBS critical region (WBSCR) (**Figure 1B** **)**. For the three genes immediately downstream of the WBS deletion (*HIP1*, *POR* and *MDH2*), we observed higher interactions with the entire WBS deletion region when compared to the region telomeric to these viewpoints. This could in part be due to spatial clustering of active gene-dense regions [31], [32] as the WBSCR contains more genes than the equidistant downstream flanking region. Even though extensive interactions were seen with the entire critical region, these three genes interact primarily with the region that includes the elastin (*ELN*), *LIMK1*, *EIF4H* and *CLIP2* genes (**Supplementary Figure S4**). We also found interactions with the centromeric low-copy repeat (LCR) region, but we cannot exclude that this merely reflects its high similarity with the nearby telomeric LCR. Alternatively, as the *HIP1, POR* and *MDH2* viewpoints are immediately adjacent to the telomeric LCR, this interaction loop might be a chromatin loop caused by the mispairing of these two repetitive and highly homologous sequences. Existence of such loop was postulated to facilitate excision and thus deletion of the intervening sequence causing WBS [33]. The centromeric genes, *ZNF107*, *ASL* and *KCTD7* that map at a greater distance of the WBSCR than the telomeric viewpoints, also loop with that genomic interval albeit not as strongly (**Figure 1B**). The *GBAS* gene located 17 Mb away from the WBSCR and on the other arm of HSA7, does not directly interact with the WBSCR.

Apart from interactions within the WBSCR, we also found significant interactions between the expression-modified genes themselves (**Figure 1B**). Many of these interactions and their relative intensities are reciprocal (i.e. the same architecture with the same intensity is identified using two different starting viewpoints). Some other interesting interacting partners shared between telomeric and centromeric viewpoints include the genes *CALN1* and *AUTS2*. Coherently, the expression of *AUTS*2 is significantly downregulated in WBS cells (**Table 1**) confirming previous results [16]. *ZNF107*, which is not significantly changed in expression in WBS patient cell lines, also interacts with some of its neighboring genes including the *HIP1*/*MDH2* region and a region within the WBSCR.

### Structural changes concurrently modify gene expression, chromatin architecture and histones marks

**Figure 2:**
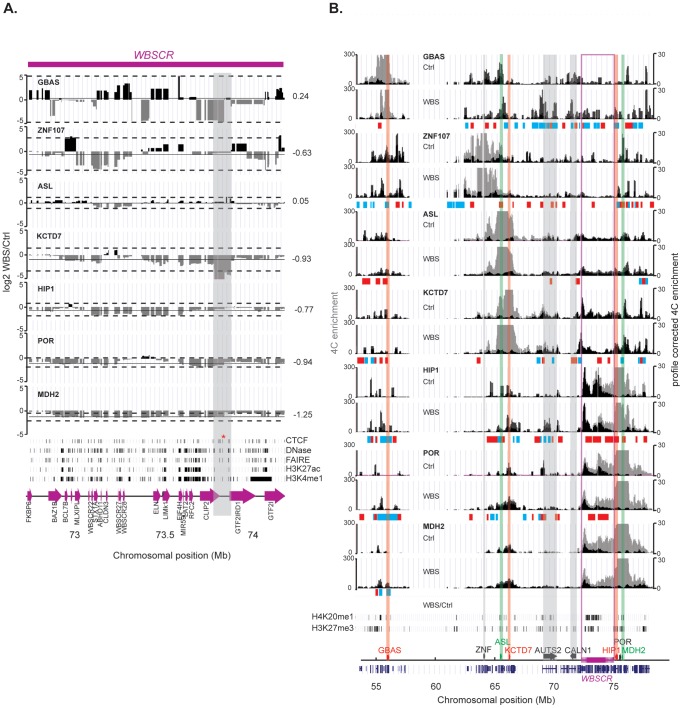
Modification of short-range interactions in WBS compared to control cells. (**A**) Close-up of the log2-fold interaction changes in WBS versus Ctrl within the WBSCR. The black line indicates the median of the changes within the WBSCR, which is also displayed at the right of each graph. The dashed lines show the 95% confidence interval. The positions of all genes are displayed at the bottom with purple arrows. The area highlighted in grey pinpoints the higher interactions in Ctrl cells between the *KCTD7* viewpoint and the region around the *CLIP2* and *GTF2IRD1* genes. The black ticks below show the location of the five marks of regulatory regions in GM12878 cells (as found in the ENCODE data), including CTCF binding sites, DHSs, FAIRE sites, H3K27ac and H3K4me1 and binding sites, with one overlapping mark highlighted with a red asterisk. (**B**) Windowed 4C signal of each of the seven viewpoints in both Ctrl and WBS cells around the WBSCR (see the legend of **Figure 1B** for details about the structures outlined). The log2-fold change of the windowed 4C data in WBS over control cells was calculated, and the resulting positive or negative Bricks are indicated below each viewpoint graphs, by blue or red bars, respectively. The significant changes in histone marks (as defined by SICER) are plotted below by ticks.

To address whether changes in expression of flanking genes upon deletion of the WBSCR are congruent with modifications in chromatin loops, we replicated the 4C assays in a lymphoblastoid cells from a female WBS patient (**Figure 2B**, **Supplementary Figures S5,S6**). Overall there is no drastic reorganization of the chromatin. In most cases, interactions are not abrogated but only modified in their intensity in cells with the 7q11.23 microdeletion consistent with the maintenance of one normal allele. From 58% (*GBAS*) to 89% (*MDH2* viewpoint), of the interacting regions are shared between the Ctrl and WBS cell lines. We next calculated changes in interaction frequency in both cell lines and determined positive and negative ratio Bricks, corresponding to interactions that are significantly increased or decreased in WBS cells, respectively (see **Supplementary Figure S2** for a detailed pipeline). We found that interactions within the WBSCR are on average decreased approximately two-fold in the WBS cells for the viewpoints mapping close to the WBSCR (*MDH2*, *POR*, *HIP1*, *KCTD7* and *ZNF107*), consistent with normal looping intensity in the remaining allele and absence of interaction in the deleted allele (**Figure 2A**). Interestingly, interactions between *KCTD7* and a region around the *CLIP2* and *GTF2IRD1* genes within the WBSCR were more than two fold diminished in WBS cells. We used the ENCODE datasets from the GM12878 lymphoblastoid cell line to search for regulatory elements within this region [29], [30], and found many regulatory marks within this region. In particular we pinpointed a CTCF binding site (highlighted with a red asterisk in **Figure 2A**), which overlaps with both marks of open chromatin (defined by DHSs and FAIRE) and a H3K4me1 binding site. As a result of the deletion, on the rearranged allele the viewpoints are positioned closer on the linear DNA molecule to the region mapping on the other side of the WBSCR. Interaction between these viewpoints and regions beyond the deletion may therefore be increased in WBS cells as previously found in the study of structural rearrangements with 4C [34]. We failed to identify such changes (**Figure 2B**), possibly because our viewpoints map too far away from the breakpoints (*HIP1* the closest viewpoint maps more than 1 Mb away). We hypothesized that only specific DNA/gene loops between regions on opposite sides of the WBSCR might be changed with the deletion, complicating the chromatin landscape. Corroboratively, in WBS cells the *GBAS* viewpoint is closer in space to the *HIP1*, *POR* and *MDH2* genes, while the *POR* viewpoint and the *AUTS2* gene interact less (**Figure 2B**). We then searched for enrichment of the six marks of regulatory chromatin taken from the ENCODE data on GM12878 cells in the differentially interacting regions. We found less consistent correlations as compared to interacting regions in Ctrl cells alone, except for enrichment of DHSs at most viewpoints (**Supplementary Table S3**), both at positive and negative ratio Bricks. In some instances, we identified interesting patterns of changes: around genes particularly, an increased interaction in WBS cells was concomitant with flanking reduction of looping intensity (**Supplementary Figure S7**). This observation suggests that chromatin reorganization is not dramatic, but rather that the intensity of long-range interactions is modified locally around certain loci. This is consistent with other work that showed that chromatin reorganization is mirrored in local changes in interactions (e.g. on the *Hox* gene clusters [25]) and that chromatin has constrained mobility [31], [35], [36].

To gain insights into the effects of a structural rearrangement on the chromatin landscape at the nucleosome level, we also monitored histone modifications on a genome-wide scale. We measured by ChIP-seq the status of H4K20me1 (monomethylation of Lysine 20 of histone H4) and H3K27me3 (trimethylation of Lysine 27 of histone H3), as proxies for open and condensed chromatin, respectively [37], in lymphoblastoids of a female patient affected by WBS, and compared them to the female Ctrl individual. We found that 4C interacting regions of the six long arm viewpoints are enriched in H4K20me1 marks compared to the rest of chromosome 7 in Ctrl cells (P = 1×10^−4^ for *ASL*, *HIP*1, *POR* and *MDH*2, P = 6×10^−4^ for *KCTD*7 and P = 4×10^−2^ for *ZNF107*, permutation test N = 10000), consistent with the clustering of open, actively transcribed regions (**Figure 1A**). H3K27me3 epigenetic marks are similarly enriched in regions interacting with the *POR* and *ASL* viewpoints (P = 1x10^−3^, permutation test N = 10000), suggesting that chromatin clustering might be determined more by the presence of genes than accessibility of the chromatin (**Figure 1A**). Overlapping islands of both open and closed chromatin marks were observed in mammalian embryonic stem cells and differentiated cells, as well as in various ENCODE cell lines [38]–[41]. These regions are defined as “bivalent domains”, in which gene promoters are in a poised state with very low levels of transcription. Significant changes in histone modifications in WBS versus Ctrl cells occurred within the WBSCR (as a result of its copy number change) but also throughout the flanking regions (**Figure 2B**). Upon close examination of the histone modifications at the expression-modified genes, we found that four of the six expression-modified genes used as viewpoints (*GBAS*, *POR, ASL* and *HIP1*) show a statistically significant change in chromatin opening between Ctrl and WBS cells (**Table 1**, difference between histone modification peaks defined by SICER with a FDR<1%, see methods for details). *GBAS* and *POR* show a decrease in H4K20me1 marks that parallel their diminished relative expression level in WBS patient cells, whereas an increase in this mark of open chromatin is seen at the *ASL* locus concomitant to its higher expression (**Table 1**). Similarly, *AUTS2* and *CALN1*, which are interaction partners of several of the studied viewpoints showed significant chromatin changes in WBS cells (FDR<1%). *HIP1* shows an increase in H4K20me1 that does not parallel its diminished expression in WBS cells. However, it also presents a significant increase in H3K27me3 marks, which parallels its change in expression (**Table 1**). *ZNF107* presents a significant decrease in H4K20me1 marks in WBS cells even though its expression is not modified in these cells. In summary, structural changes may induce concurrent changes in gene expression, chromatin architecture and histones marks.

## Discussion

Structural variants have been shown to capture 10% to 25% of the expression variance [17], [42]. They influence gene expression by modifying gene dosage and altering the expression of normal-copy number genes located in their vicinity [5], [6], [15], [16], [43]. This effect can be long range with changes in expression of genes positioned megabases away [19], [23]. We investigated the underlying mechanism of genome organization by combining high-throughput chromosome conformation capture and chromosome-wide profiling of histone modifications. Our results suggest that structural rearrangements can influence expression levels of flanking normal-copy genes in part by affecting large-scale chromatin conformation in various ways.

First, deletion of specific long-range regulatory elements within the rearrangement, such as enhancers and/or boundary elements, can cause changes in their finely tuned regulatory function and thus in the expression of their target genes. Concordantly, we detect alterations of intrachromosomal interactions (chromosomal looping) between expression-affected gene loci and the rearranged interval in WBS cells using chromosome conformation capture. Some of these alterations go beyond the expected two-fold decrease. Specifically, we observe that the interaction between the *KCTD7* viewpoint and the region between *CLIP*2/*GTF2IRD1* is abolished in WBS cells rather than diminished by 50%, suggesting allele-specific chromatin interaction, which was recently postulated by studying the inactive X chromosome [44]. We infer that chromosome looping can be allelically biased through allele-specific regulatory activity and/or gene expression [45]–[47]. Interestingly, a number of regulatory marks are located within this region in the corresponding GM12878 lymphoblastoid cell line monitored by the ENCODE project. One particular mark of open chromatin (detected by both DNase hypersensitivity and FAIRE) in this region coincides with H3K4me1 modifications, an enhancer mark, but also CTCF binding.

Second, in addition to modifying specific *cis*-acting DNA regulatory elements, a structural rearrangement could also exert its effect on gene expression by changing the overall chromatin topology and DNA accessibility. Genes might be co-regulated by clustering into “chromatin globules” independently of functional relationship [48]. A strong correlation between interaction frequency and the position of DNase I hypersensitivity sites shows that the organization of the chromatin is tightly linked to the accessibility to regulatory factors [31], [49], [50]. Dislocation of a spatially clustered set of genes might disrupt or modify specific position effect as well as chromatin accessibility, and thereby affect the expression of these genes – even if this clustering is driven by gene density. Consistent with these assumptions, we observed frequent interactions between the normal-copy genes flanking the WBSCR and the critical interval itself. The identified chromatin interactions are modified in cells from WBS patients, suggesting that changes in the genome structure cause concomitant modifications of chromatin interactions and histone marks. The complexity of the observed changes prevents us to distinguish whether the changes are a primary or secondary effect of the mechanisms described above. The observed changes are however not restricted to genes that show significant expression changes in WBS cells as we also observe chromatin architecture and histone marks modifications of the *ZNF107* locus hinting that other mechanisms must also be at play.

Some of these modifications may be associated with specific phenotypic features observed in genomic disorders patients. A tantalizing example from our study is the *AUTS2* gene. Its looping architecture, chromatin structure changes and expression modification in WBS cells designate this gene as a potential candidate in some of the phenotypes shown by WBS or WBRdupS patients. *AUTS2* is mutated or translocated in autistic patients and individuals with intellectual disabilities [51]–[53], phenotypes shared by patients with Williams-Beuren region duplication syndrome. Even though the lymphoblastoid cell lines used in this study might not be the best target cell/tissue for many of the genes involved in these disease processes, experiments with these cells are still worth pursuing, simply because we cannot exclude a broad to ubiquitous expression pattern for these genes. Of note previous experiments have shown a high degree of correlation in gene expression levels between different tissues/cell lines for the genes mapping within the aneuploid segments [16], [54]. Further studies are warranted to confirm that *AUTS*2 expression is modified in other tissues.

## Materials and Methods

All lymphoblastoid cell lines used in this study were collected with written appropriate informed consent and approval of the local ethics committee (i.e. "Commission cantonale vaudoise d'éthique de la recherche sur l'être humain http://www.unil.ch/Jahia/site/fbm/op/edit/pid/36053), made exception of the WBS (GM13472) and Control (Ctrl, GM07006) lines that were obtained from the Coriell Institute for Medical Research Biobank (http://www.coriell.org/).

### Cells

Cells were grown in RPMI 1640 medium (Gibco) with addition of 10% fetal calf serum and 1% penicillin-streptomycin. The rearrangement was examined by array CGH using Human CGH 3x720K whole-genome tiling array (Nimblegen) following the manufacturer's protocol. Known changes in the expression levels of *GBAS, ASL, KCTD7, HIP1, POR* and *MDH2* in WBS patient cell lines were confirmed in GM13472 relative to the Ctrl cell line cells by Taqman real-time quantitative PCR using previously published primers pairs and probes [16].

### Circularized Chromosome Conformation Capture – sequencing (4C-seq)

The 4C-seq assay was performed as described in [55] and based on the 4C protocol developed by [32], [56]. Briefly, GM07006 (Ctrl) and GM13472 (WBS) lymphoblastoid cell lines were grown at 37°C. 5×10^7^ exponentially growing cells were harvested and crosslinked with 1% formaldehyde, lysed and cut with the restriction enzyme *Bgl*II. After ligation and reversal of the crosslinks, the DNA was purified to obtain the 3C library. This 3C library was further digested with *Nla*III and circularized to obtain a 4C library. The inverse PCR primers to make the 4C-seq templates were designed to contain the Illumina adaptor tails, as well as the bait-specific sequences for each of the seven loci we interrogated. The list of primers is described in **Supplementary Table S1**. The seven viewpoints were selected at the *Bgl*II fragment containing the transcriptional start sites of four genes located upstream of the WBSCR (*GBAS* 16.7 Mb, *ZNF107* 8.8 Mb, *ASL* 7.6 Mb, and *KCTD7* 7 Mb upstream respectively), and three other genes located immediately downstream of the WBSCR (*HIP1* 0.7 Mb, *POR* 0.96 Mb and *MDH2* 1 Mb downstream respectively). For the three nearby downstream viewpoints, we amplified at least 0.6 µg of 4C template, whereas for the further away upstream viewpoints, we amplified at least 1 µg of 4C template (using about 100 ng per inverse PCR reaction). We multiplexed the 4C-seq templates by pooling the samples in equimolar ratios in two sets, representing 3 viewpoints each (*POR*, *KCTD7* and *GBAS* in one set and *ASL*, *MDH2* and *HIP1* in the second set). Replicate 4C libraries were prepared for both the Ctrl and the WBS cell lines. We randomly selected three viewpoints (*ASL*, *POR* and *MDH2*) and replicated the experiments. All 4C-seq multiplexed samples were analyzed on a Illumina GAIIx flow-cell using a 76-bp single-end sequencing run. These studies were completed with a 4C assay with viewpoint mapping at the transcriptional start site of *ZNF107*. This gene did not show any significant change in expression in WBS versus Control cell lines [16]. This additional 4C-seq library was prepared from the same 4C template and run on a 100-bp single-end Illumina HiSeq flow cell.

### 4C-seq data analysis

4C-seq data were analyzed as described in [55]. Briefly, the multiplexed samples were separated, undigested self-ligated reads removed, and the reads mapped to a virtual library of *BgIII* fragments. Reads were then normalized to the total number of reads. A running mean algorithm was applied to smooth the data (19 fragments per window). As the data from the three replicated viewpoints were strongly correlated (**Supplementary Figure S1**), we used the average of each data point for these experiments. To remove the strongly interacting local “background” region, we modeled the data to apply a profile correction similar to the one described in [26] using a fit with a slope -1 in a log-log scale [31]. We used a domainogram algorithm to detect significantly interacting regions without imposing a fixed window size [24]. The positive signals were ranked per chromosome and Bricks (Blocks of Regulators In Chromosomal Kontext) were called based on a FDR threshold of 0.1 for “short-range” interactions, defined as interactions within 2.5 Mb up- and downstream of *GBAS* and *MDH2*, the first and last viewpoint, respectively (HSA7 coordinates: 53,532,296–78,116,172; about 25 Mb around the WBSCR). As long-range interactions are more prone to random ligation events, we used a more stringent FDR threshold of 0.001 for the genomic space outside of these borders (called the “long-range” region). Interacting regions were then defined by merging consecutive Bricks. To determine differentially interacting regions between the WBS and Ctrl cells, we first computed the log2 ratio of WBS over Ctrl of the smoothed profile corrected data and selected ratio Bricks that were specific to either WBS or Ctrl (**Supplementary Figure S2**). To assess the significance of those regions we quantified the number of reads inside each Brick, and averaged over consecutive Bricks within each region. We then compared the distribution of the (Ctrl+WBS) log counts in these regions versus Bricks outside by a Wilcoxon rank-sum test. We obtained very significant p-values (ASL: 2.4e–28, GBAS: 8.4e–78, HIP1: 4.3e–74, KCTD7: 4.1e–54, MDH2: 6.2e–18, POR: 1.3e–46 and ZNF: 3.6e–80), indicating that selected ratio Bricks contained a significantly higher number of reads than all Bricks. 4C data are deposited under accession number GSE33867.

To estimate if the long-range interactions of the seven viewpoints were significantly enriched in genes or histone modifications we performed permutation tests (n = 10000) with all RefSeq genes or histone modified regions identified by SICER with a FDR = 1×10^−4^. To permute the interacting regions we used shuffleBed from BEDtools version 2.10.1 [57]. For comparison with expressed genes only, we used the published ENCODE expressing datasets from the lymphoblastoid cell line GM12878 which is similar to our Ctrl cells (wgEncodeCaltechRnaSeqGm12878R2x75Th1014Il200SigRep1V4rep1 and wgEncodeCaltechRnaSeqGm12878R2x75Th1014Il200SigRep2V4 [58]). To search for correlation of our interacting maps with regulatory elements, we also used the ENCODE database Specifically, we used the ChIP-seq peaks called on chr7 for six marks: CTCF, p300, H3K4me, H3K27ac binding sites, as well as regions of open chromatin defined by DNase HS and FAIRE

### Chromatin Immunoprecipitation - sequencing (ChIP-seq)

Crosslinking was performed by adding formaldehyde solution (Sigma Aldrich) to the cells in growth medium to a final concentration of 0.5%. After 5-minute incubation at room temperature, cross-linking agent was quenched with glycine. 1×10^6^ cells were centrifuged and used directly in the ChIP assay. Cells were lysed by addition of cell lysis buffer (1% SDS, EDTA, Tris-HCl pH 8.1) and 10- minute incubation on ice. Next, chromatin was sheared using a Bioruptor sonicator (Diagenode) at medium power settings (30 seconds on – 30 seconds off cycles for 45 minutes). Sonication efficiency was tested by reversing cross-links of a chromatin sample and running the obtained DNA on a 1.5% agarose gel. Fragmented chromatin was used directly in the ChIP assay or frozen at −80°C for latter usage.

ChIP was performed as suggested in [59]. Briefly, chromatin was diluted 10-fold in ChIP dilution buffer (0.01% SDS, 1.1% Triton X100, 1.2 mM EDTA, 16.7 mM Tris-HCl pH 8.1, 167 mM NaCl) and subsequently immunoprecipitated using antibodies raised against H3K27me3 (Millipore 07-449) and H4K20me1 (Abcam ab9051). The antibody-histone complex was collected using magnetic beads (Invitrogen). Beads were washed twice with dialysis buffer (2 mM EDTA, 50 mM Tris-HCl pH 8.0, 0.2% sarcosyl) and four times with wash buffer (100 mM Tris-HCl pH 9.0, 500 mM LiCl, 1% NP40, 1% sodium deoxycholate). DNA was then eluted and the crosslinks reversed. Following RNase A and proteinase K treatments, samples were purified using DNA purification kit (Qiagen). The concentration of DNA was measured using a Qubit instrument (Invitrogen) and 10 ng of each sample was used for library preparation. Enrichment of the precipitated DNA was assessed by comparing the levels of DNA corresponding to known open and closed chromatin regions by quantitative PCR. Primer pairs corresponding to exon 2 of *GAPDH* and intron 5 of the *GRM8* gene were used for the H4K20me1 and H3K27me3 ChIP, respectively. The same primer pairs were used reciprocally as negative controls.

Sequencing libraries of immunoprecipitated DNA samples were prepared as described by the manufacturer (Illumina) and then sequenced on two lanes of an Illumina GAIIx flow-cell each (single end, 36mer tags). Sequencing reads were mapped to the human reference genome (hg19, GRCh37) using Bowtie algorithm allowing 2 mismatches and no seed [60]. Duplicates potentially arisen were removed, i.e. only a single tag was retained from identical sequences [61]. Note that in the remaining analyses, we only considered uniquely matching tags, i.e. between 21.7 and 32.1×10^6^ and 3.3 and 15.3×10^6^ for H4K20me1 and H3K27me3, respectively.

The identification of ChIP-enriched regions was performed assuming a Poisson distribution of the tag counts by using SICER [62] version 1.1 with two libraries (SICER-df-rb.sh) and the following parameters: window size 200 bp, gap size 400 bp, for H4K20me1 and gap size 600 bp for H3K27me3 as suggested by the package authors, and E-value 100. We selected candidate islands with a FDR = 1×10^−4^ defined by SICER for the Ctrl and the rearranged sample and further used these islands to assess statistical significance of differential modification of a given region using the DEseq package [63] which assumes a negative binomial distribution of the tag counts. As a positive control, we verified the change in ChIP-tags in the rearranged interval and found a correlation between the decrease in ChIP-tags and the two-fold lower copy number of the deleted region. To identify genes that were significantly altered in their chromatin status - and thus possibly also in expression - we screened the chromatin changes of RefSeq genes defined according to the genomic coordinates [64]. ChIP-seq data are deposited under accession number GSE33784.

## Supporting Information

Figure S1 **Reproducibility of 4C-seq experiments.** (**A**) Mirror plot of the windowed 4C scores of two biologically independent replicates using *MDH2* as viewpoint (Pearson correlation  = 0.97). (**B**) Overview of the number of mappable reads per viewpoint and per cell line, as well as Pearson correlation coefficient between bioreplicates.
(PDF)

Figure S2 **Steps followed to generate the ratios BRICKS.** To allow the identification of BRICKS with a negative log2 ratios, we run the domainogram algorithm by sorting the data on an ascending order, which puts the high negative ratios on top position of the initial ranking. The two sets of BRICKS have been treated independently, in the following way: 1) selecting and grouping consecutive BRICKS as described in the material & methods, 2) removing BRICKS found in both datasets (for overlaps greater than 5%), 3) removing genomics gaps (UCSC, hg19) from BRICKS and 4) excluding BRICKS that were not part of a selected BRICKS in either Ctrl or WBS BRICKS. Finally both sets were grouped together to form a unique set of BRICKS.
(PDF)

Figure S3 **Heatmap showing the percent coverage of HSA7 by Bricks of each viewpoint, as well as the percent of HSA7 that overlaps between Bricks of the different viewpoints, indicating that the viewpoint interactions cluster by their linear chromosomal position.**
(PDF)

Figure S4 **Close-up of the interactions of the seven viewpoints with the WBSCR in cells from a healthy control individual.** The two areas highlighted in grey show the strongly interacting regions at the LCRcen (centromeric LCR) and the region within WBSCR. Pink boxes indicate the mapping of genes within the WBSCR.
(PDF)

Figure S5 **Interactions of seven genes on HSA7 in cells from a WBS patient.** Windowed 4C signal of each of the seven viewpoints along the entire chromosome. The black ticks below each graph show the location of the Bricks. The density of genes is shown at the bottom. Areas highlighted in blue pinpoint some examples of strong correlation of gene-dense regions with H4K20me1 marks and highly interacting regions. The mapping of the viewpoints and the WBSCR is indicated at the bottom.
(PDF)

Figure S6 **Close-up of the interactions of the seven viewpoints with the WBSCR in cells from a WBS patient.** The two areas highlighted in grey show the strongly interacting regions at the LCRcen (centromeric LCR) and the region within WBSCR. Pink boxes indicate the mapping of genes within the WBSCR.
(PDF)

Figure S7 **Examples of regions with modified interactions with the** ***POR*** **viewpoint.** The y-axis represents postprocessed normalized counts. The log2-fold change of the windowed 4C data in WBS over Ctrl cells is plotted. Positive or negative Bricks are indicated below each viewpoint graph, by blue or red bars, respectively. In WBS cells, the region around the *CDK6* gene (**A**) or sonic hedgehog (*SHH* gene) (**B**) interacts with the *POR* gene, whereas in Ctrl cells, the flanking regions interact more frequently, indicating local changes in interactions.
(PDF)

Table S1 **4C-seq primer sequences.**
(PDF)

Table S2 **Overview of the location of Bricks per viewpoint for control and WBS cells, as well as the ratio.**
(PDF)

Table S3 **Correlation analyses between six different marks of regulatory elements and interacting regions in Ctrl cells (Ctrl Bricks), in all differential interacting regions significantly decreased (negative ratio Bricks) or increased (positive ratio Bricks) in WBS versus Ctrl cells.** Permutation test with number of permutation  = 1000. Significant p-values are highlighted in grey.
(PDF)
