# Supplementary material for: Structural Variation-Associated Expression Changes Are Paralleled by Chromatin Architecture Modifications
Source: PLoS One. 2013 Nov 12;8(11):e79973. doi: 10.1371/journal.pone.0079973 (PMC3827143; doi:10.1371/journal.pone.0079973)
Supplement: Figure S1 — Reproducibility of 4C-seq experiments. (A) Mirror plot of the windowed 4C scores of two biologically independent replicates using MDH2 as viewpoint (Pearson correlation = 0.97). (B) Overview of the number of mappable reads per viewpoint and per cell line, as well as Pearson correlation coefficient between bioreplicates. (PDF) [file pone.0079973.s001.pdf]

A.

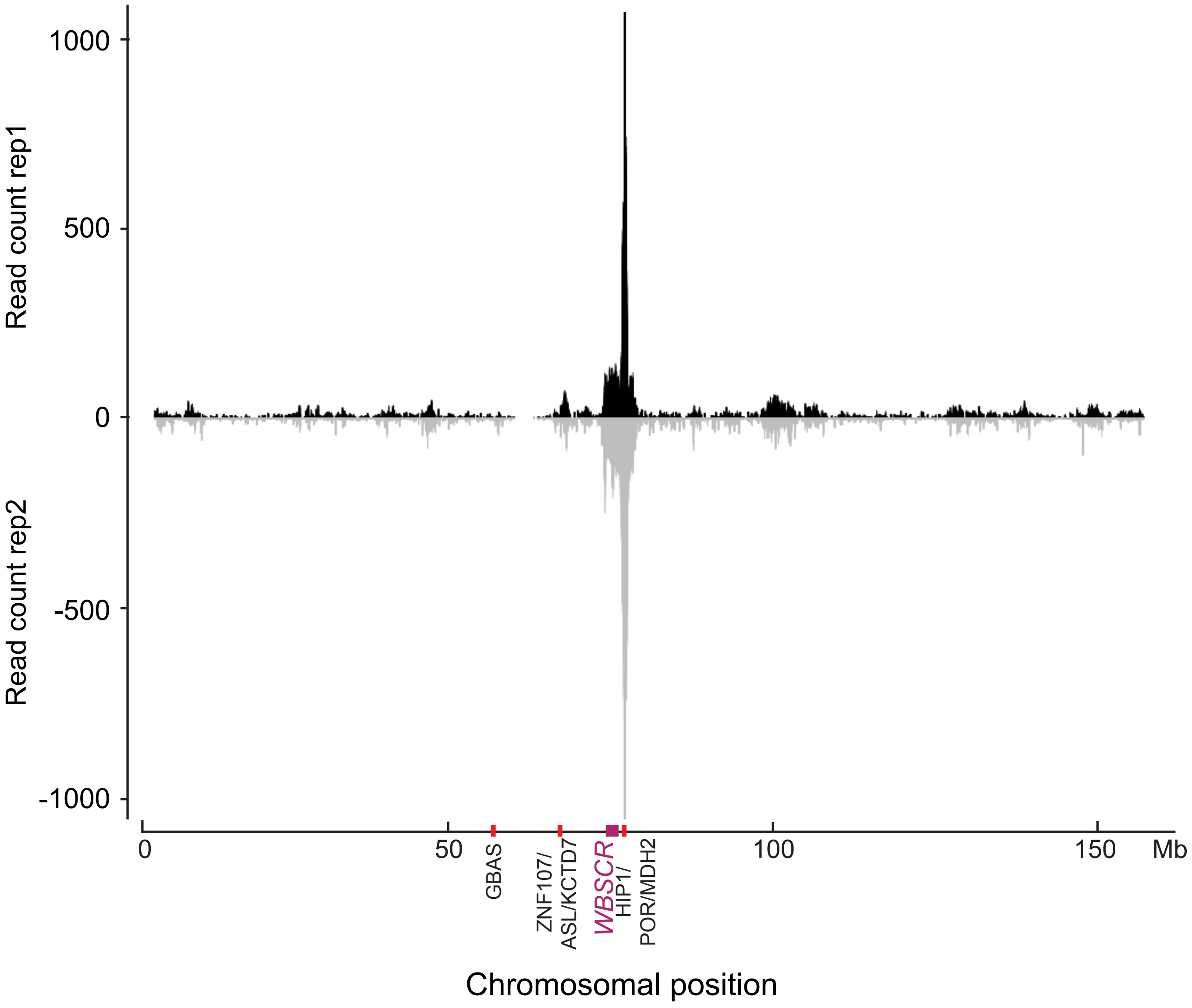

B.

Viewpoint	Cells	Replicate	# mappable reads (million)	correlation (Pearson) btw replicates smoothed data
GBAS	WT	R1	2.65	
	WBS	R1	2.05	
ZNF107	WT	R1	19.5	
	WBS	R1	51.5	
ASL	WT	R1	6.33	0.83
		R2	4.38	
	WBS	R1	6.4	0.93
		R2	3.74	
KCTD7	WT	R1	2.4	
	WBS	R1	1.45	
HIP1	WT	R1	2.03	
	WBS	R1	1.26	
POR	WT	R1	3.17	0.96
		R2	2.52	
	WBS	R1	5.1	0.93
		R2	6.05	
MDH2	WT	R1	3.07	0.97
		R2	7.38	
	WBS	R1	2.73	0.96
		R2	3.88	
