# Supplementary figures and images for: Structural Variation-Associated Expression Changes Are Paralleled by Chromatin Architecture Modifications
Source: PLoS One. 2013 Nov 12;8(11):e79973. doi: 10.1371/journal.pone.0079973 (PMC3827143; doi:10.1371/journal.pone.0079973)

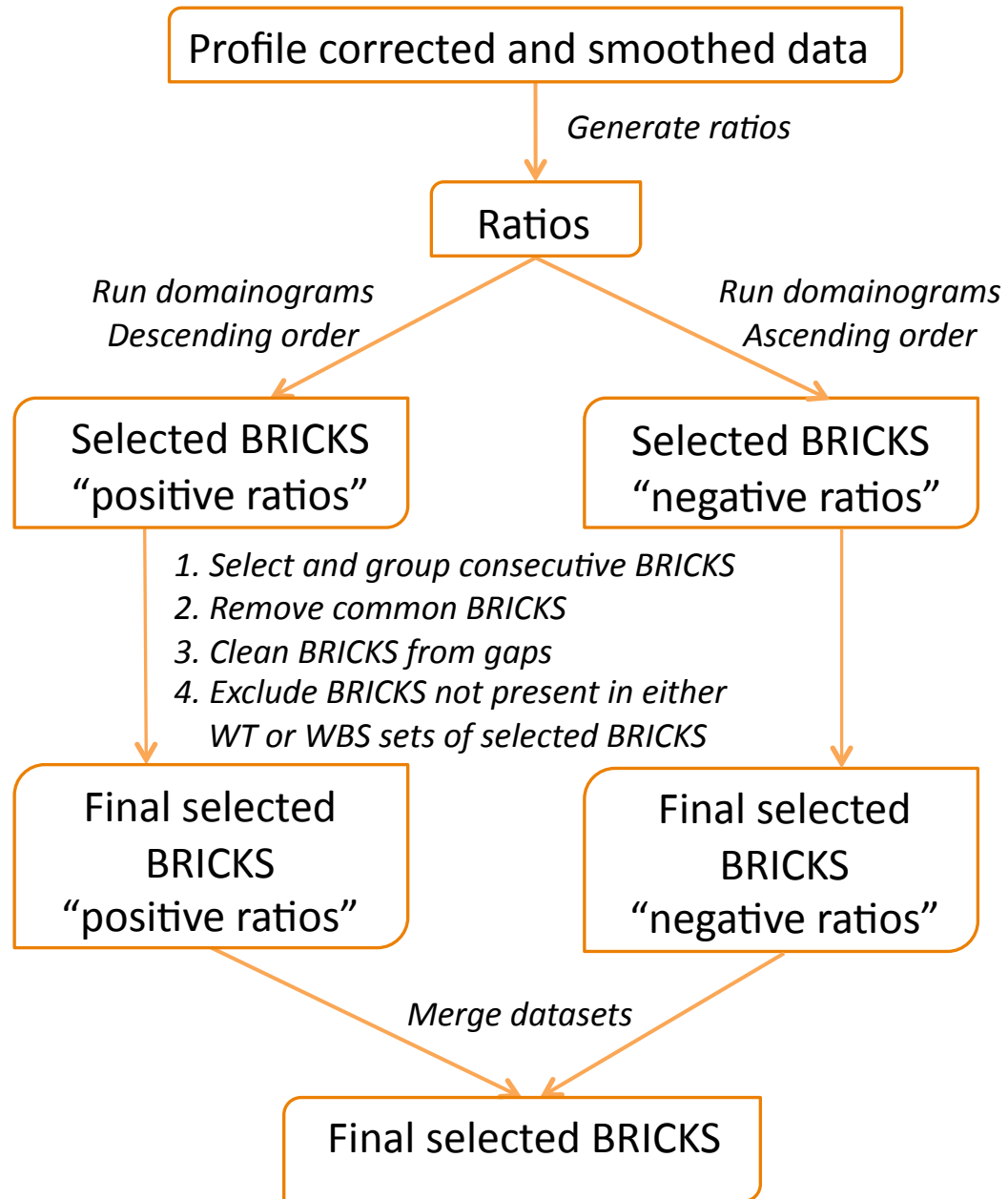

Supplement: Figure S2 — Steps followed to generate the ratios BRICKS. To allow the identification of BRICKS with a negative log2 ratios, we run the domainogram algorithm by sorting the data on an ascending order, which puts the high negative ratios on top position of the initial ranking. The two sets of BRICKS have been treated independently, in the following way: 1) selecting and grouping consecutive BRICKS as described in the material & methods, 2) removing BRICKS found in both datasets (for overlaps greater than 5%), 3) removing genomics gaps (UCSC, hg19) from BRICKS and 4) excluding BRICKS that were not part of a selected BRICKS in either Ctrl or WBS BRICKS. Finally both sets were grouped together to form a unique set of BRICKS. (PDF) [file pone.0079973.s002.pdf]

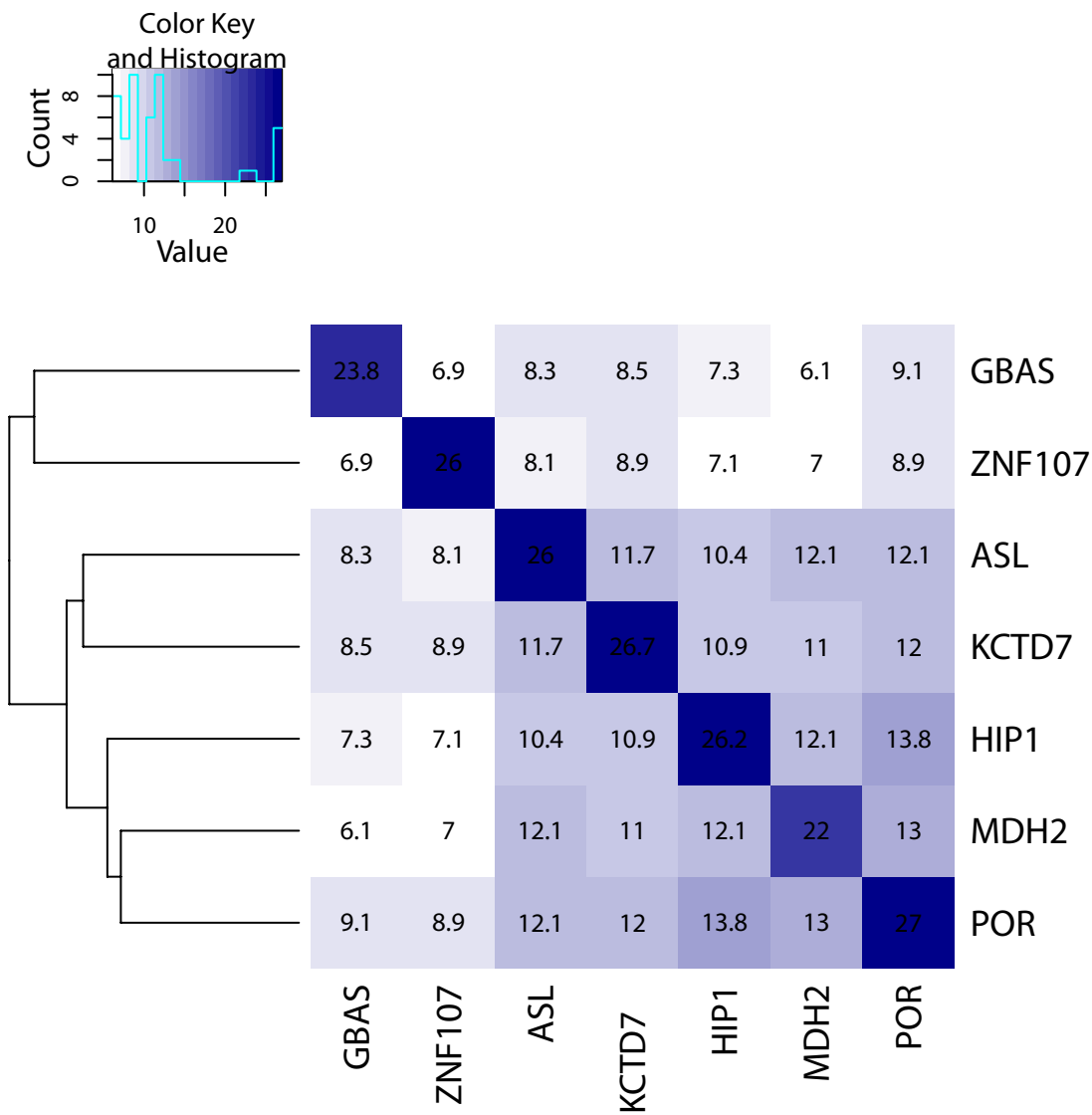

Supplement: Figure S3 — Heatmap showing the percent coverage of HSA7 by Bricks of each viewpoint, as well as the percent of HSA7 that overlaps between Bricks of the different viewpoints, indicating that the viewpoint interactions cluster by their linear chromosomal position. (PDF) [file pone.0079973.s003.pdf]

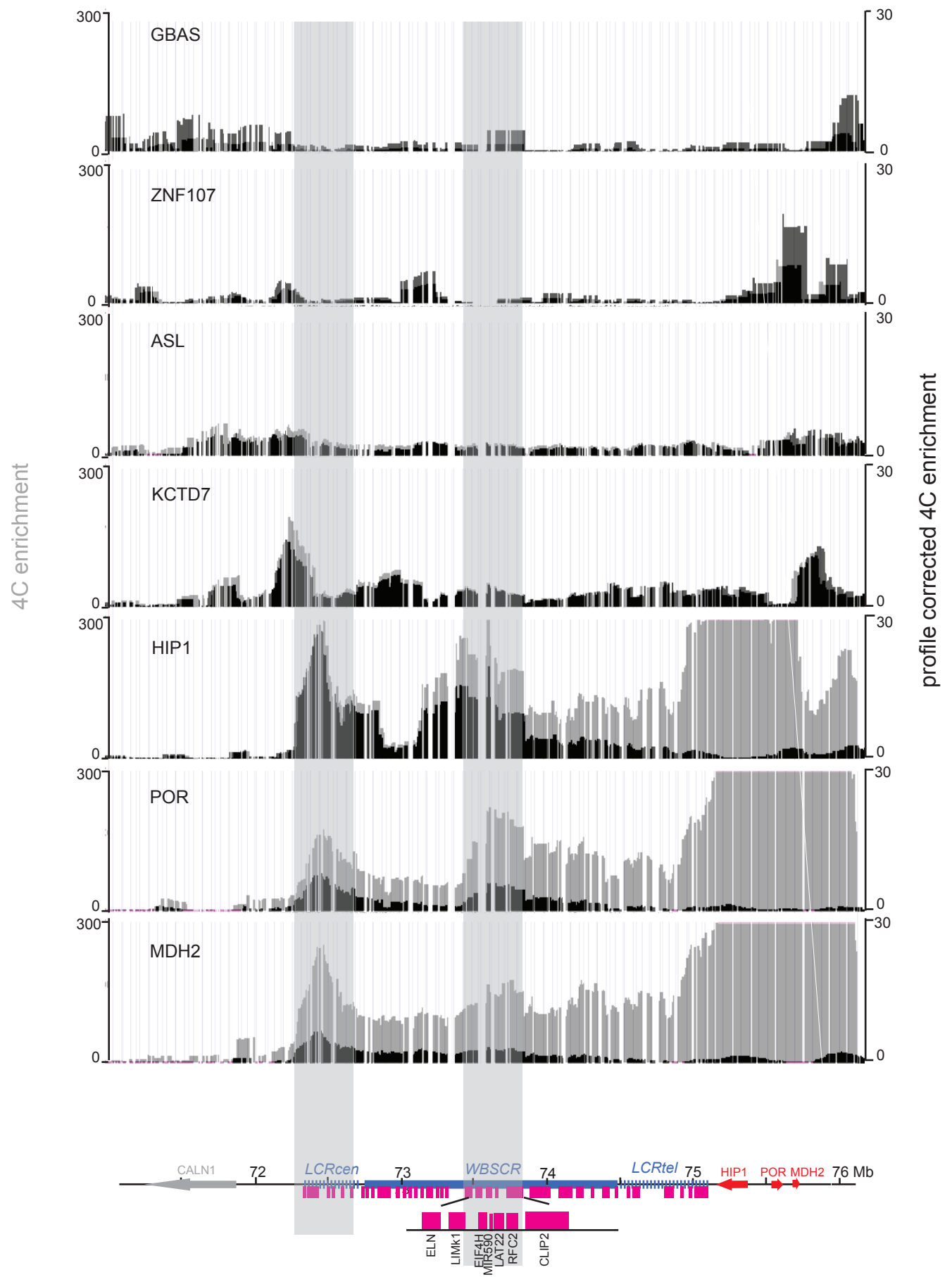

Supplement: Figure S4 — Close-up of the interactions of the seven viewpoints with the WBSCR in cells from a healthy control individual. The two areas highlighted in grey show the strongly interacting regions at the LCRcen (centromeric LCR) and the region within WBSCR. Pink boxes indicate the mapping of genes within the WBSCR. (PDF) [file pone.0079973.s004.pdf]

Supplementary Figure S5

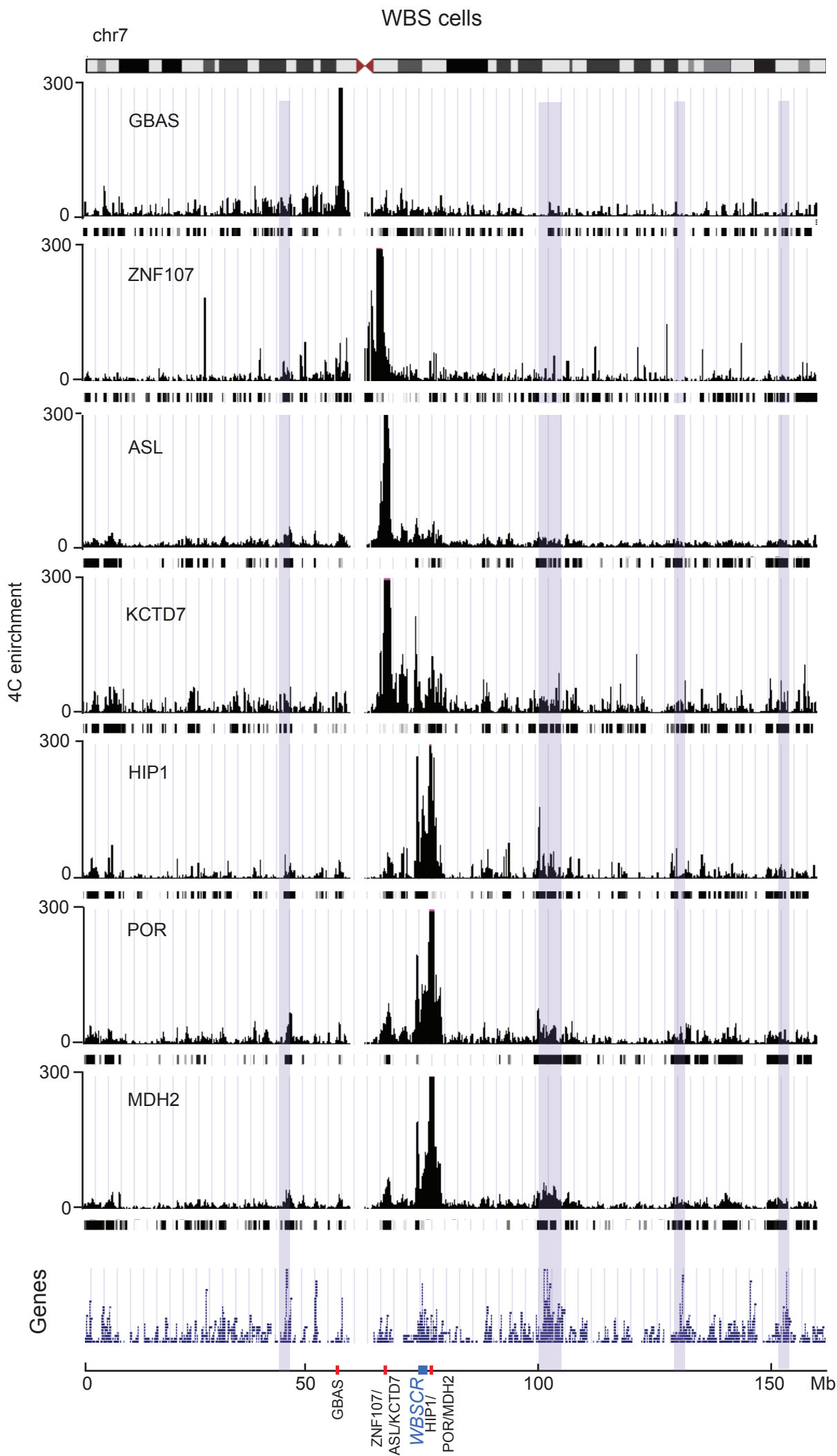

Supplement: Figure S5 — Interactions of seven genes on HSA7 in cells from a WBS patient. Windowed 4C signal of each of the seven viewpoints along the entire chromosome. The black ticks below each graph show the location of the Bricks. The density of genes is shown at the bottom. Areas highlighted in blue pinpoint some examples of strong correlation of gene-dense regions with H4K20me1 marks and highly interacting regions. The mapping of the viewpoints and the WBSCR is indicated at the bottom. (PDF) [file pone.0079973.s005.pdf]

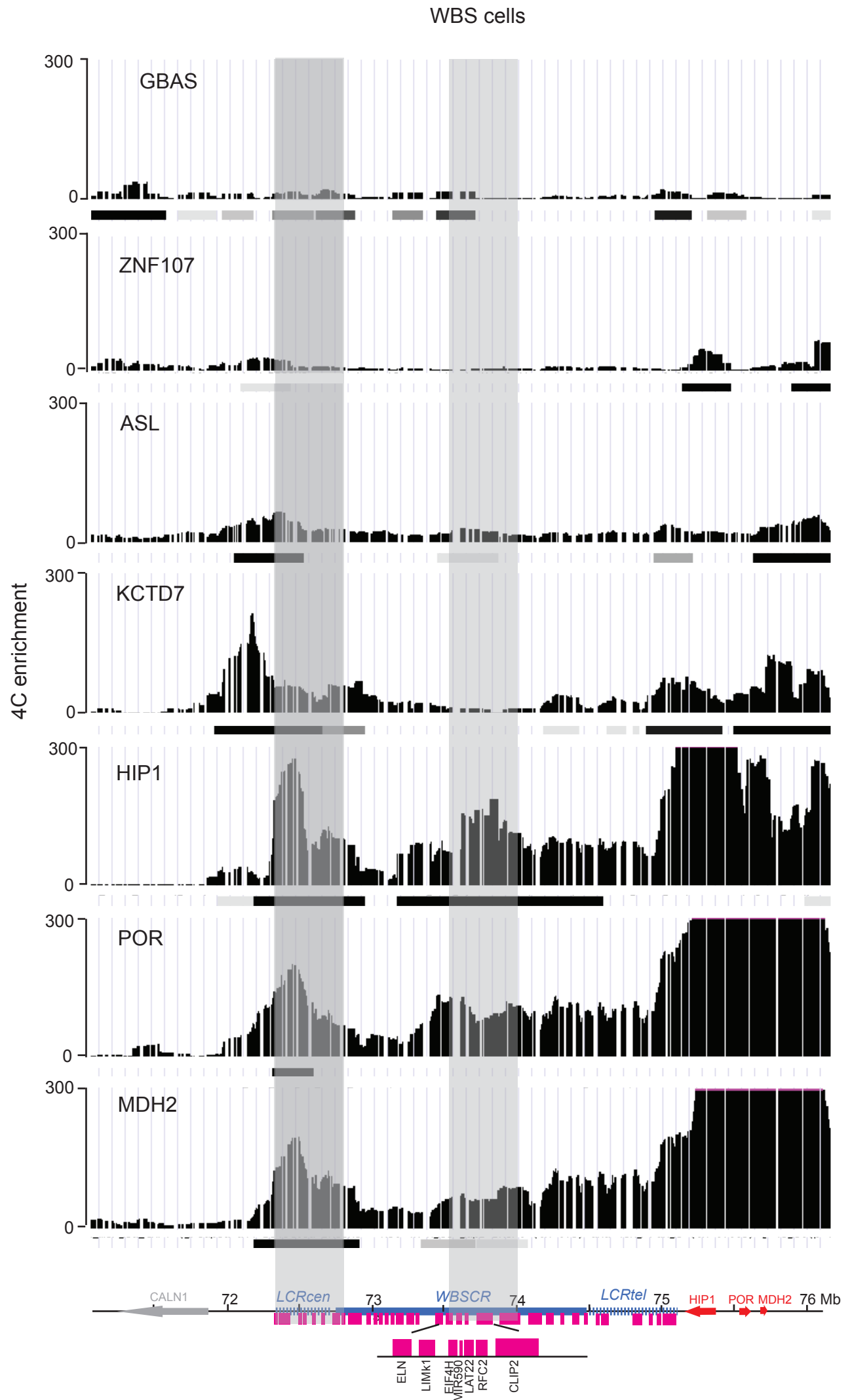

Supplement: Figure S6 — Close-up of the interactions of the seven viewpoints with the WBSCR in cells from a WBS patient. The two areas highlighted in grey show the strongly interacting regions at the LCRcen (centromeric LCR) and the region within WBSCR. Pink boxes indicate the mapping of genes within the WBSCR. (PDF) [file pone.0079973.s006.pdf]

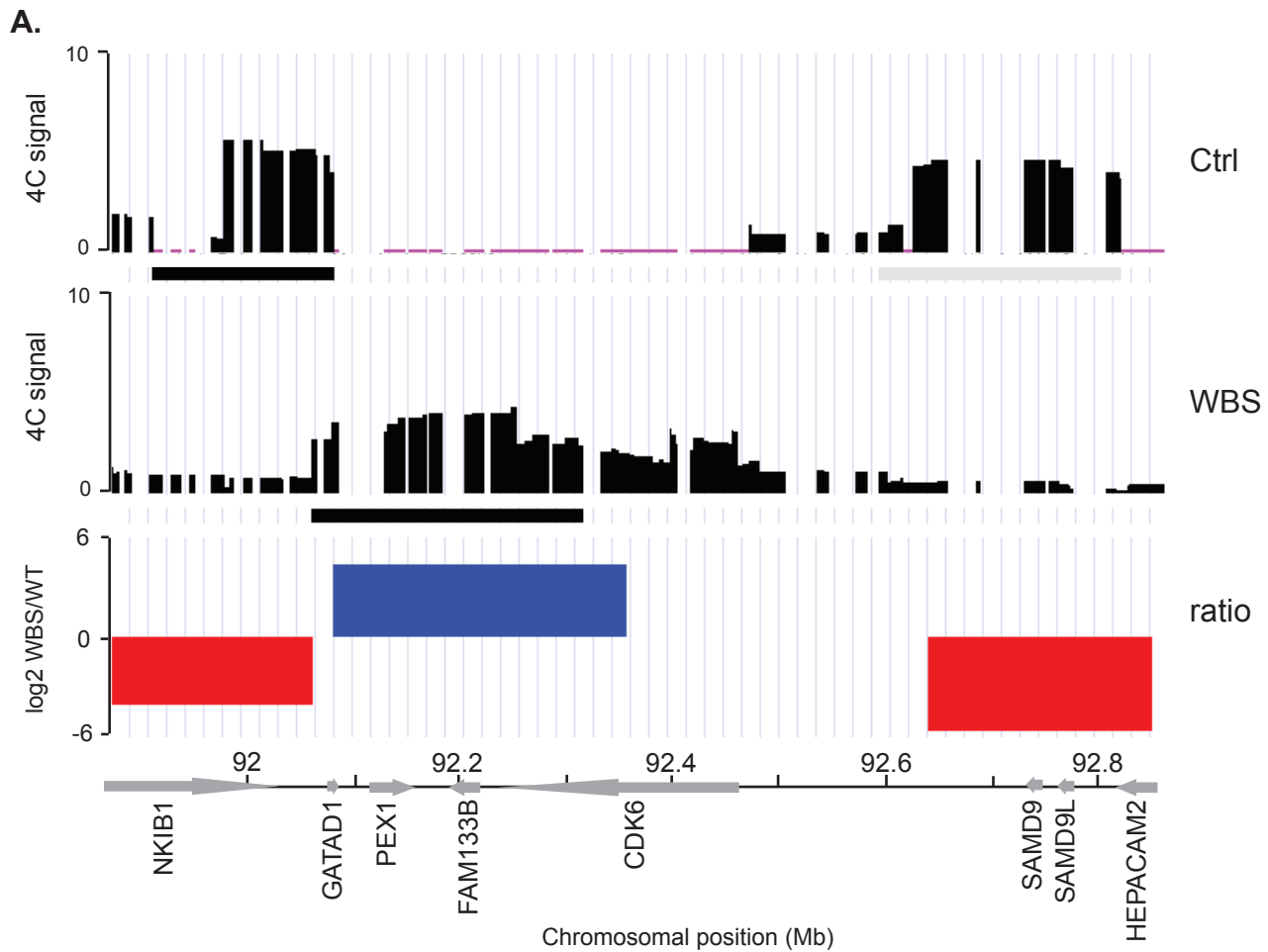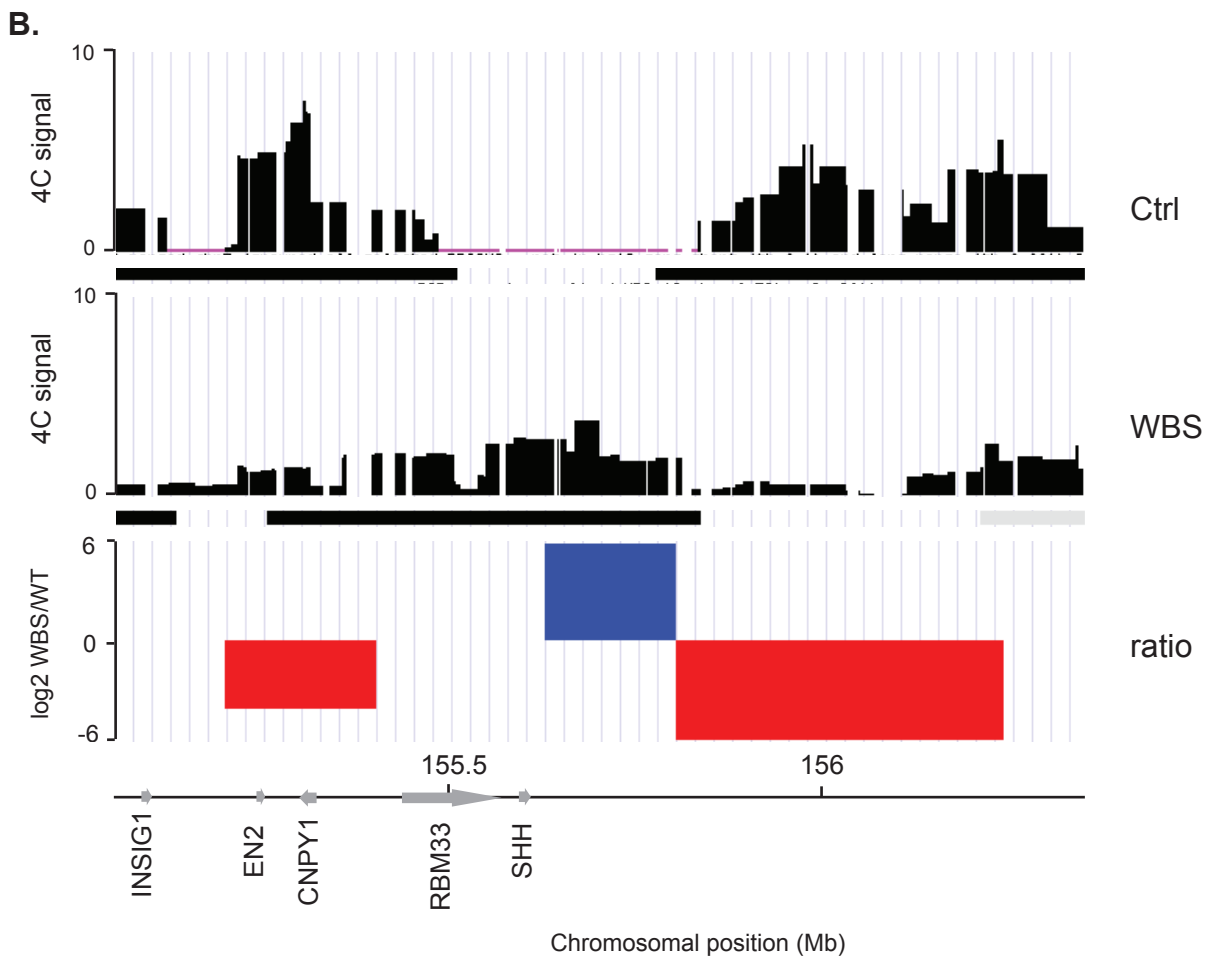

Supplement: Figure S7 — Examples of regions with modified interactions with the POR viewpoint. The y-axis represents postprocessed normalized counts. The log2-fold change of the windowed 4C data in WBS over Ctrl cells is plotted. Positive or negative Bricks are indicated below each viewpoint graph, by blue or red bars, respectively. In WBS cells, the region around the CDK6 gene (A) or sonic hedgehog (SHH gene) (B) interacts with the POR gene, whereas in Ctrl cells, the flanking regions interact more frequently, indicating local changes in interactions. (PDF) [file pone.0079973.s007.pdf]
