# Supplementary material for: Structural Variation-Associated Expression Changes Are Paralleled by Chromatin Architecture Modifications
Source: PLoS One. 2013 Nov 12;8(11):e79973. doi: 10.1371/journal.pone.0079973 (PMC3827143; doi:10.1371/journal.pone.0079973)
Supplement: Table S1 — 4C-seq primer sequences. (PDF) [file pone.0079973.s008.pdf]

Supplementary Table S1. 4Cseq primer sequences

Gene viewpoint	Primer name	Sequence
GBAS	GBAS_4C_seq_F	AATGATACGGCGACCACCGAACACTCTTTCCCTACACGACGCTCTTCCGATCT TTCTAAGCGGACATTTTCCT
	GBAS_4C_seq_R	CAAGCAGAAGACGGCATACGAGTGTGGTGATTCATCCCTGT
ZNF107	ZNF107_4c_seq_F	AATGATACGGCGACCACCGAACACTCTTTCCCTACACGACGCTCTTCCGATCTTGCAACAGAAGGAAATACCAAC
	ZNF107_4C_seq_R	CAAGCAGAAGACGGCATACGATCTCTCATTCCCCTTCCATC
ASL	ASL_4C_seq_F3	CAAGCAGAAGACGGCATACGA GCTCCAGTGATCAGGACCAG
	ASL_4C_seq_R3	AATGATACGGCGACCACCGAACACTCTTTCCCTACACGACGCTCTTCCGATCT TGGGTTGAATGAGCAACAGT
KCTD7	KCTD7_4C_seq_F	AATGATACGGCGACCACCGAACACTCTTTCCCTACACGACGCTCTTCCGATCT CT TTCAGAGCTCACCAAGGTTTG
	KCTD7_4C_seq_R	CAAGCAGAAGACGGCATACGACCAGCCACGTACTCTGAAAA
HIP1	HIP1_4C_seq_R	AATGATACGGCGACCACCGAACACTCTTTCCCTACACGACGCTCTTCCGATCT GTAGTGAGCGGGGGCATT
	HIP1_4C_seq_F	CAAGCAGAAGACGGCATACGAGTTGGGCACCTGCTTCAT
POR	POR_4C_seq_F	AATGATACGGCGACCACCGAACACTCTTTCCCTACACGACGCTCTTCCGATCTAGTCTCTTCCCCTCCCTACCAC
	POR_4C_seq_R	CAAGCAGAAGACGGCATACGATACGTAAGGAACGCGTCCAA
MDH2	MDH2_4C_seq_F2	AATGATACGGCGACCACCGAACACTCTTTCCCTACACGACGCTCTTCCGATCTCTGATACTGGAGCCCGAGATGA
	MDH2_4C_seq_R	CAAGCAGAAGACGGCATACGACCCAGTTCTGTTAGGCGTTC
