# Supplementary material for: Structural Variation-Associated Expression Changes Are Paralleled by Chromatin Architecture Modifications
Source: PLoS One. 2013 Nov 12;8(11):e79973. doi: 10.1371/journal.pone.0079973 (PMC3827143; doi:10.1371/journal.pone.0079973)
Supplement: Table S2 — Overview of the location of Bricks per viewpoint for control and WBS cells, as well as the ratio. (PDF) [file pone.0079973.s009.pdf]

Supplementary Table S2. Overview of the location of Bricks per viewpoint for control and WBS cells, as well as the ratio.

POR VIEWPOINT

Control cells

	start coord	end coord	Value
Br1	15217	232484	6
Br2	282484	396453	6
Br3	428259	635654	6
Br4	825985	1915801	6
Br5	1965476	2275159	6
Br6	2552804	2869130	6
Br7	3387015	3562514	6
Br8	4493382	4801374	4.38
Br9	5099888	6696557	6
Br10	7465215	7655819	6
Br11	7676716	7821057	6
Br12	9171809	9331481	4.16
Br13	11860561	12167911	6
Br14	14548442	14647660	5.61
Br15	15251401	15418182	6
Br16	17201925	17349272	5.12
Br17	20086555	20250628	4.22
Br18	20261178	20400514	4.02
Br19	21803797	22001706	6
Br20	22221874	22734498	6
Br21	24070903	24770122	6
Br22	24824078	25196244	6
Br23	25220763	25549586	6
Br24	25696003	25816152	3.3
Br25	26375223	26731167	6
Br26	28428856	28659479	6
Br27	29194667	29348419	6
Br28	30217904	31018743	5.94
Br29	31110433	31243948	5.74
Br30	32487435	32830925	5.99
Br31	32856563	33059063	4.28
Br32	33316564	33592737	5.9
Br33	35782666	36366208	6

WBS cells

	start coord	end coord	Value
BrW1	429002	655927	4.56
BrW2	747875	3021187	6
BrW3	3086762	3586566	6
BrW4	3773661	4008132	5.84
BrW5	4250585	4448857	5.21
BrW6	5020861	6770262	6
BrW7	7093403	7286101	4.49
BrW8	8044798	8272841	5.33
BrW9	8467618	8590905	3.53
BrW10	8838248	8943508	3.45
BrW11	9149606	9469893	6
BrW12	15901745	16111707	4.89
BrW13	16885721	17060112	4.56
BrW14	17182806	17549291	6
BrW15	17593211	17762622	5.7
BrW16	20213141	20521852	5.98
BrW17	22044896	22193434	4.9
BrW18	22268881	22992645	6
BrW19	23012359	23161758	3
BrW20	23329069	23544812	5.13
BrW21	24269248	24460180	3.62
BrW22	24865458	25005764	6
BrW23	25687649	25865279	5.84
BrW24	26103157	26240248	5.91
BrW25	26447979	26657626	3.77
BrW26	26873870	27062233	4.48
BrW27	27401207	27547910	3.81
BrW28	27745595	27918675	6
BrW29	28749246	28846010	3.17
BrW30	29544150	30087251	6
BrW31	30159011	30564769	6
BrW32	30805035	31009812	5.04
BrW33	35108823	35302796	3.08

Ratio BRICKs

	start coord	end coord	Value
BrR1	2866906	3352301	6
BrR2	3440026	3576908	-5.21
BrR3	3576908	4116635	6
BrR4	4137312	4560296	6
BrR5	4554027	4815852	-6
BrR6	6308133	6587758	-5.81
BrR7	7303976	7761266	-6
BrR8	8117925	8317244	6
BrR9	8467611	8641914	6
BrR10	8858172	9040924	5.76
BrR11	9216199	9412837	5.26
BrR12	11805958	12149252	-6
BrR13	14456253	14795556	-6
BrR14	15093148	15531453	-6
BrR15	16076727	16623045	6
BrR16	16807984	17110581	6
BrR17	17545147	17880070	6
BrR18	20499052	20823248	-5.69
BrR19	21484554	21818580	6
BrR20	21808129	22001713	-6
BrR21	22078054	22193434	5.24
BrR22	22208137	22331669	-6
BrR23	22353592	22433550	3.88
BrR24	22666354	23162105	5.77
BrR25	23542107	23917603	3.25
BrR26	24181731	24740033	-6
BrR27	25005973	25189869	-4.61
BrR28	25202983	25624719	-6
BrR29	25961653	26240248	5.81
BrR30	26255991	26447972	-3.94
BrR31	26695598	26890456	6
BrR32	26899868	27089576	6
BrR33	27402977	27549091	6

Br34	37236242	37393627	4.04	BrW34	35816703	36013263	4.32	BrR34	27667693	27992169	6
Br35	37473634	38032589	6	BrW35	36210559	36480885	3.11	BrR35	28316684	28447190	6
Br36	39407446	39505902	5.8	BrW36	36923175	37021820	4.78	BrR36	28469574	28624642	-6
Br37	39630330	39802793	6	BrW37	37047841	37520213	6	BrR37	28658233	29181909	6
Br38	39961372	40177086	6	BrW38	38142600	38317453	6	BrR38	29194667	29348419	-6
Br39	40942588	41109041	5.04	BrW39	39203618	39433697	6	BrR39	29607671	29996122	6
Br40	43329718	43724391	6	BrW40	39584788	40221589	6	BrR40	30684883	30921456	-3.32
Br41	43746925	44294642	6	BrW41	42968090	43138422	4.4	BrR41	30938757	31122520	5.57
Br42	44428400	45183265	6	BrW42	43827770	45571267	6	BrR42	31122526	31255860	-4.1
Br43	45422467	45708834	6	BrW43	46150725	46333780	4.23	BrR43	32086043	33077119	-6
Br44	45735243	45952292	5.96	BrW44	47153640	47266091	3.9	BrR44	33280730	33527267	-6
Br45	47208963	47304297	3.31	BrW45	50170553	50370631	4.67	BrR45	35939639	36337620	-5.82
Br46	47592838	47822926	5.59	BrW46	50410631	50518785	4.67	BrR46	36896721	36995631	4.24
Br47	48942300	49104748	3.14	BrW47	53256073	53371348	5.67	BrR47	37047841	37355869	6
Br48	50270915	50370631	4.25	BrW48	54961931	55106679	2.32	BrR48	37359080	37416996	4.35
Br49	50410631	50459488	4.25	BrW49	55394593	55941084	6	BrR49	37416996	38043536	-6
Br50	51218960	51394051	4.56	BrW50	56661596	56948566	2.12	BrR50	38250139	38416219	5.89
Br51	53825357	54004869	2.82	BrW51	64444482	64880360	2.24	BrR51	39142066	39462503	6
Br52	55009657	55189785	6	BrW52	65103825	66809707	6	BrR52	39462503	39505902	-4.85
Br53	55290378	55474127	2.04	BrW53	67361567	67571818	1.78	BrR53	39531634	39708842	-4.01
Br54	56986274	57219753	2.51	BrW54	69628378	69767884	1.35	BrR54	39707542	39893927	6
Br55	62919556	63118767	1	BrW55	72174189	72891545	6	BrR55	40152401	40568769	-6
Br56	64401111	64644708	1.78	BrW56	73321593	73686975	2.36	BrR56	40942587	41209602	-5.88
Br57	65185368	65422951	2.87	BrW57	73701399	74039613	1.04	BrR57	43245388	43724391	-6
Br58	65606299	65882096	1.37	BrW58	92061814	92315360	3.89	BrR58	44999834	45306815	5.79
Br59	65912647	66436168	6	BrW59	98407827	99169158	6	BrR59	45697330	45927745	-6
Br60	66545209	66772585	1.53	BrW60	99382170	100086815	6	BrR60	46142473	46361635	6
Br61	68051989	68182884	3.31	BrW61	100202923	100556043	6	BrR61	47621537	47848834	-4.12
Br62	68795634	69186940	5.88	BrW62	100606043	100706295	6	BrR62	48942300	49104748	-5.38
Br63	69609162	69703157	1.12	BrW63	101378373	101619905	5.91	BrR63	49979841	50291280	6
Br64	69989874	70091201	1.06	BrW64	101659032	102517538	6	BrR64	50442559	50537311	5.33
Br65	70156131	70334775	1.66	BrW65	104496668	105162916	6	BrR65	53264552	53371348	5.53
Br66	72210531	74456275	6	BrW66	105689315	106259199	6	BrR66	53825357	54187439	-5.96
Br67	74524671	74702466	1.19	BrW67	106972616	107255497	5.07	BrR67	54978639	55067588	3.6
Br68	75139249	75386775	1.31	BrW68	110750925	110852193	5.18	BrR68	55067595	55262846	-5.99
Br69	75858537	76097132	1.01	BrW69	113117040	113364502	3.24	BrR69	55319130	56626740	6
Br70	86587929	86765486	4.21	BrW70	114035577	114113549	3.28	BrR70	56661596	56948566	1.45
Br71	87439982	87632488	3.41	BrW71	120649067	120772612	3.99	BrR71	56989854	57220291	-4.65
Br72	91912392	92081874	5.36	BrW72	121730181	121907849	3.51	BrR72	62889657	63118767	-1.31
Br73	92593717	92819758	5.05	BrW73	122080616	122196369	3.33	BrR73	64346314	64444475	-1.07

Br74	95527913	95701182	6
Br75	95765514	95947756	6
Br76	97139510	97321713	4.23
Br77	98324294	98564761	5.59
Br78	98795463	100483533	6
Br79	100659181	101196733	6
Br80	101604578	102748521	6
Br81	103177662	103343214	4.6
Br82	104349183	104503045	4.03
Br83	104715815	105287641	6
Br84	105306594	106410081	6
Br85	106493310	107359157	5.87
Br86	107513886	107988903	6
Br87	108024048	108215050	5.16
Br88	111933299	112127457	6
Br89	117691758	117858683	3.99
Br90	123269915	123354621	3.33
Br91	127256773	127826258	6
Br92	127901972	129515964	6
Br93	129615283	129996030	5.9
Br94	130783534	130962710	4.29
Br95	131646749	131807380	4.7
Br96	132431823	132517237	4.1
Br97	132623547	132804355	6
Br98	133017325	133165917	3.9
Br99	134663798	134874234	4.92
Br100	135425334	135581182	4.8
Br101	138061560	138206334	3.35
Br102	138260733	138521855	4.4
Br103	139171817	139379377	5.83
Br104	139404377	139712065	5.83
Br105	139846762	140323563	6
Br106	141019887	141417907	6
Br107	141713282	141996524	5.92
Br108	144350380	144654861	5.93
Br109	147833465	148238083	6
Br110	148327705	148735251	3.62
Br111	149031927	149575540	6
Br112	149882107	150432659	6
Br113	150848973	151410321	6

BrW74	126776643	126908650	6
BrW75	127594965	127737102	4.12
BrW76	127885149	128382285	6
BrW77	128485564	128646720	3.07
BrW78	128705905	129007410	4.43
BrW79	129126297	129391466	6
BrW80	129478021	129912854	6
BrW81	130605006	131725463	6
BrW82	133060506	133360804	5.61
BrW83	134342878	134483354	4.25
BrW84	134540031	134971930	5.84
BrW85	135056655	135245638	6
BrW86	135282013	135427668	5.63
BrW87	136850805	137068297	3.08
BrW88	138599078	139345588	6
BrW89	139527770	140384925	6
BrW90	140535191	140899259	6
BrW91	141089378	141650197	6
BrW92	141657634	141827467	3.8
BrW93	142398502	142538487	6
BrW94	144271129	144364323	4.72
BrW95	147712961	147851048	6
BrW96	148238090	149175653	6
BrW97	149205820	149661395	5.93
BrW98	149941482	150485978	5.68
BrW99	150619620	151443408	6
BrW100	151709116	152426663	6
BrW101	154504103	155136622	6
BrW102	155260762	155838841	6
BrW103	156214449	156438311	4.8
BrW104	156568088	156852272	3.6
BrW105	156906518	157315349	6
BrW106	158394639	158611996	6
BrW107	158680384	158871171	5.98
BrW108	158970435	159127921	4.96

BrR74	64644715	64920007	2.66
BrR75	65103825	65196675	1.08
BrR76	67326518	67571818	4.23
BrR77	68051990	68182884	-4.91
BrR78	68607082	68820522	1.04
BrR79	68815433	69185378	-6
BrR80	69185378	69226200	1.1
BrR81	69989874	70091201	-1.22
BrR82	70173836	70334775	-1.78
BrR83	72672131	73241870	-1.76
BrR84	73501019	74339676	-2.16
BrR85	86626967	86811894	-6
BrR86	87470634	87644234	-6
BrR87	91874167	92061807	-4.14
BrR88	92081880	92356336	4.4
BrR89	92640061	92848325	-5.81
BrR90	95404756	95927526	-6
BrR91	97106924	97522493	-5.99
BrR92	99171083	99381165	-6
BrR93	99426456	99769759	-3.59
BrR94	100075299	100276410	-5.93
BrR95	102517545	102748521	-4.62
BrR96	103112524	103422496	-6
BrR97	104290384	104465559	-5.98
BrR98	104540952	104758877	5.82
BrR99	104959613	105575565	-6
BrR100	106021921	106302254	-3.27
BrR101	107522725	108294008	-6
BrR102	110650431	110887430	5.94
BrR103	111838572	111966275	3.06
BrR104	111966282	112146527	-5.55
BrR105	112919064	113156841	4.15
BrR106	113187627	113566961	5.53
BrR107	113821456	114061432	6
BrR108	121631875	122301357	6
BrR109	123270488	123362000	-3.35
BrR110	126779546	126973155	6
BrR111	127310805	127656753	-6
BrR112	127768754	127967333	3.64
BrR113	128561296	128709496	-5.14

Br114	151810335	152008620	4.69
Br115	152573750	152791658	5.95
Br116	154439141	154813593	6
Br117	154907631	155513175	6
Br118	155779717	156466718	6
Br119	156483855	156649057	6
Br120	157770096	158179901	6
Br121	158486615	158707091	6

BrR114	129062726	129260915	-4.06
BrR115	129514108	129704969	6
BrR116	130712214	131745504	6
BrR117	132541175	132924017	-6
BrR118	133106280	133389005	6
BrR119	134348572	134504848	5.37
BrR120	135023511	135362711	6
BrR121	135370493	135638177	-6
BrR122	136769398	137203383	5.83
BrR123	137834172	138402239	-6
BrR124	138646139	138928084	5.29
BrR125	139652742	139818980	5.66
BrR126	140583763	141018663	6
BrR127	141013794	141140533	-4.65
BrR128	141312991	141516467	5.06
BrR129	141781182	142014676	-5.97
BrR130	142391237	142514398	4.11
BrR131	144172071	144330094	5.91
BrR132	144375797	144729625	-6
BrR133	147631272	147763423	5.97
BrR134	147851048	148081425	-5.82
BrR135	148142878	148242751	-6
BrR136	149713402	149956468	6
BrR137	152424483	152638933	-6
BrR138	152740560	153165805	5.54
BrR139	154448254	154612570	-5.96
BrR140	155203212	155405137	-4.14
BrR141	155632851	155807737	5.78
BrR142	155807737	156242886	-6
BrR143	156517258	156698254	-6
BrR144	156852279	157463838	5.61

ASL VIEWPOINT

Control cells

	start coord	end coord	Value
Br1	179233	232484	3.74
Br2	282484	431782	3.74
Br3	541256	842144	6
Br4	1197066	2444355	6
Br5	2542909	2920903	3.62
Br6	2929074	3188765	5.26
Br7	3659242	3818162	3.06
Br8	3941757	4149268	5.62
Br9	5086389	5368883	3.16
Br10	5982852	6938350	6
Br11	12416972	12624302	3.76
Br12	14348937	14554259	3.57
Br13	17093234	17321955	6
Br14	17833616	17952737	5.23
Br15	18558847	18713331	4.07
Br16	22522610	23013187	6
Br17	23512815	24182978	6
Br18	24213329	24486833	6
Br19	25174883	25326451	5.11
Br20	27254041	27395015	3.91
Br21	28227873	28455109	4.49
Br22	29283529	29676949	5.98
Br23	29793334	30253350	6
Br24	30310708	30521296	6
Br25	30843083	31060373	3.87
Br26	35625479	35750138	3.43
Br27	35865387	36118527	5.16
Br28	36955073	37087153	6
Br29	37247480	37402907	4.25
Br30	37841668	38020938	6
Br31	38832611	39078127	6
Br32	39471449	39864505	5.78
Br33	40061834	40468824	5.8

WBS cells

	start coord	end coord	Value
BrW1	52737	232484	3.72
BrW2	282484	313570	3.72
BrW3	463121	1474266	6
BrW4	1648599	3406038	6
BrW5	4556457	7272863	6
BrW6	7736023	8267258	6
BrW7	17178498	17358095	6
BrW8	20566988	20731021	3.67
BrW9	21391519	21639239	6
BrW10	22241811	22591586	6
BrW11	22639168	23089193	6
BrW12	23362847	23694677	6
BrW13	24847455	25191733	6
BrW14	26117716	26572865	5.96
BrW15	26644361	26918685	6
BrW16	28074415	28248838	3.95
BrW17	29982596	30776397	6
BrW18	35313898	35496889	3.75
BrW19	35654145	35798557	4.69
BrW20	36124431	36684556	6
BrW21	37116293	37267906	4.08
BrW22	38329342	38537539	4.77
BrW23	39625134	39864505	3.44
BrW24	39867615	40148096	5.25
BrW25	41821842	41984670	3.66
BrW26	43535232	45375476	6
BrW27	47443864	47647626	6
BrW28	50042328	50301413	6
BrW29	55535210	55725448	3.34
BrW30	55979844	56227193	2.17
BrW31	72039875	72509963	5.89
BrW32	73432201	73841286	1.13
BrW33	74905050	75168284	2.69

Ratio BRICKs

	start coord
BrR1	38727
BrR2	209711
BrR3	282484
BrR4	886267
BrR5	3587823
BrR6	4773918
BrR7	6660719
BrR8	7185980
BrR9	7385882
BrR10	7910809
BrR11	12516742
BrR12	14507822
BrR13	17086934
BrR14	17234243
BrR15	17711430
BrR16	18659896
BrR17	20402602
BrR18	21625480
BrR19	22208136
BrR20	22929452
BrR21	23678378
BrR22	24130186
BrR23	24329573
BrR24	25174883
BrR25	26088650
BrR26	26644361
BrR27	27270014
BrR28	27866307
BrR29	28348425
BrR30	29275748
BrR31	29441404
BrR32	35220440
BrR33	36892802

Br34	42957426	43354448	4.4	BrW34	75588598	76207805	6	BrR34	37114085
Br35	43469046	44422704	6	BrW35	76523607	76657474	1.96	BrR35	37296443
Br36	44479053	45540100	6	BrW36	76942869	77121651	5.22	BrR36	37758417
Br37	47292426	47526186	4.58	BrW37	77459137	77596251	2.59	BrR37	37891401
Br38	54135292	54434030	6	BrW38	86632785	87072756	6	BrR38	38829217
Br39	55086111	55459526	3.13	BrW39	87518015	87900077	3.98	BrR39	39471448
Br40	55538414	56164565	4.37	BrW40	89893888	90114244	3.58	BrR40	40061835
Br41	64365222	64653910	1.35	BrW41	90120271	90339628	5.02	BrR41	41855159
Br42	69399294	69609155	1.48	BrW42	91674440	91935678	4.1	BrR42	47251709
Br43	69806794	69915759	1.01	BrW43	92032830	92437763	6	BrR43	47526193
Br44	71584338	72382144	4.73	BrW44	92856516	93093108	4.66	BrR44	53965828
Br45	73102983	73385610	1.21	BrW45	98249897	99366241	6	BrR45	55098627
Br46	74803037	75084574	1.22	BrW46	99382170	100469014	6	BrR46	55697749
Br47	75452712	76077910	5.01	BrW47	100799012	101098717	4.03	BrR47	69505590
Br48	76573155	76721889	1.87	BrW48	101333067	101619905	5.48	BrR48	71680947
Br49	77124396	77402153	5.24	BrW49	101979246	102419219	5.93	BrR49	76934525
Br50	78264992	78464155	3.92	BrW50	102937275	103112517	3.18	BrR50	77130208
Br51	82385664	82547752	3.85	BrW51	103175910	103343214	5.7	BrR51	77459137
Br52	86710339	87111155	5.01	BrW52	104525506	105383546	6	BrR52	78336025
Br53	87621103	87953890	6	BrW53	106190737	106829160	6	BrR53	90137235
Br54	89911918	90126109	3.66	BrW54	107049513	107328474	4.08	BrR54	91557309
Br55	91484655	92420586	6	BrW55	107513886	107757925	5.98	BrR55	91888172
Br56	92980498	93142631	6	BrW56	107952431	108146380	4.84	BrR56	93048316
Br57	93693327	93877608	6	BrW57	112387234	112585701	3.4	BrR57	93602615
Br58	94122047	94342475	4	BrW58	116409228	116835453	5.88	BrR58	98176537
Br59	98020554	99163817	6	BrW59	117963282	118065543	3.03	BrR59	99163823
Br60	99722507	100011324	3.35	BrW60	120003894	120160945	3.48	BrR60	100458901
Br61	100612018	100820933	5.93	BrW61	120507804	120775454	6	BrR61	100606043
Br62	101981713	102419851	4.04	BrW62	123322646	123453343	6	BrR62	100801507
Br63	102559091	102861109	4.64	BrW63	126412674	126589449	6	BrR63	103112524
Br64	104693344	104905297	4.57	BrW64	126703801	126777478	3.67	BrR64	103337355
Br65	105344795	106589534	6	BrW65	126877196	127190258	5.72	BrR65	105216623
Br66	106618089	106821199	6	BrW66	127914661	128368737	5.93	BrR66	105407451
Br67	107558139	108000191	5.81	BrW67	128597519	129083947	6	BrR67	105789222
Br68	110470508	110760461	5.93	BrW68	129422979	129884055	6	BrR68	106519692
Br69	111702953	111847191	3.42	BrW69	130537215	130712207	4.15	BrR69	107661467
Br70	111940566	112124177	5.11	BrW70	131181084	131418175	3.12	BrR70	110548372
Br71	112387234	112585701	6	BrW71	132574814	132915461	5.87	BrR71	111708149
Br72	112744049	112853127	5.93	BrW72	134413478	134940251	5.87	BrR72	111931714
Br73	116056872	116752355	6	BrW73	135027862	135413947	5.72	BrR73	112759161

Br74	120243151	120397801	3.96
Br75	121147635	121371524	5.99
Br76	123646124	123798379	4.65
Br77	127949822	128227865	4.77
Br78	128386280	129076491	6
Br79	129097348	129800887	6
Br80	130573989	130721877	5.89
Br81	131125950	131565144	6
Br82	131921891	132072544	6
Br83	132606609	132889727	5.77
Br84	133415684	133559341	6
Br85	134056350	134392188	6
Br86	134895487	135056648	4.25
Br87	135482041	135657690	3.58
Br88	137519012	137753115	5.87
Br89	138402609	138928084	6
Br90	139846762	140378766	6
Br91	140668750	141032916	6
Br92	141039318	141392997	5.81
Br93	141891679	142041635	5.9
Br94	142245162	142276197	6
Br95	142326197	142628040	6
Br96	142688407	143284933	5.85
Br97	144436762	144761489	6
Br98	144840610	144947178	3.15
Br99	147446009	147618921	5.48
Br100	147827610	148017321	5.79
Br101	148184696	149759098	6
Br102	149834088	150416318	6
Br103	150567842	150731287	3.05
Br104	151125443	152706050	6
Br105	154190275	154270634	5.92
Br106	154370634	154492003	5.92
Br107	154740414	155658082	6
Br108	155762684	155998250	6
Br109	156007353	157309130	6
Br110	157552380	157799212	6

BrW74	135801696	135987968	4.54
BrW75	137450784	137746950	5.79
BrW76	138711403	138991539	6
BrW77	139135663	139379377	6
BrW78	139404377	140440045	6
BrW79	140447390	140831048	5.91
BrW80	141330146	141535693	4.26
BrW81	142179519	142276197	5.66
BrW82	142326197	142383500	5.66
BrW83	142722465	143007023	6
BrW84	143828078	144148264	3.37
BrW85	144276610	144432991	6
BrW86	148144552	148315656	5.24
BrW87	148392968	149413069	6
BrW88	149721918	149958837	3.81
BrW89	150025301	150368224	6
BrW90	150557831	152608740	6
BrW91	153999207	154172066	6
BrW92	154526291	154879603	5.76
BrW93	154934468	155743032	6
BrW94	155761727	155997217	3.39
BrW95	156230783	157387884	6

BrR74	116084390
BrR75	117376561
BrR76	119080666
BrR77	120657369
BrR78	120895922
BrR79	121181602
BrR80	123269915
BrR81	126197732
BrR82	127748352
BrR83	128485564
BrR84	129186131
BrR85	129876248
BrR86	130412958
BrR87	130710152
BrR88	131095599
BrR89	131305512
BrR90	131522643
BrR91	131954418
BrR92	132884768
BrR93	133288895
BrR94	133828448
BrR95	134447771
BrR96	135845850
BrR97	137406145
BrR98	137746523
BrR99	138359974
BrR100	139135663
BrR101	139404377
BrR102	140497017
BrR103	140758651
BrR104	141413344
BrR105	141673075
BrR106	142143558
BrR107	142326197
BrR108	142363388
BrR109	144026258
BrR110	144475456
BrR111	147259943
BrR112	148242750
BrR113	149361411

BrR114	150721457
BrR115	151463315
BrR116	152617938
BrR117	153871521
BrR118	154524261
BrR119	154900149
BrR120	155886640
BrR121	157262903

end coord	Value
179232	6
232484	-3.66
431782	-3.66
1327606	4.5
3861438	-6
5086382	5.57
7199829	6
7361335	-6
7744788	5.2
8155979	4.22
12890397	-6
15129156	6
17248729	-6
17413753	6
17886741	-6
18911363	-6
21159111	6
21761730	-5.04
22489579	6
23103423	4.46
24094469	-3.13
24244804	3.6
24516304	-6
25326451	-3.42
26506921	6
26799717	3.89
27456401	-4.55
28131981	5.88
28546417	6
29387039	-6
29646375	-5.79
35528149	5.99
37116286	-5.89

MDH2 VIEWPOINT

Control cells

	start coord	end coord	Value
Br1	362134	1759281	6
Br2	2549402	3161840	6
Br3	4943000	5210594	3.96
Br4	5245043	6756865	6
Br5	7410588	7761266	6
Br6	22871772	23014021	5.09
Br7	23241073	23586626	6
Br8	24303081	24469187	4.02
Br9	24604570	24918084	6
Br10	24943176	25118296	3.84
Br11	25951338	26118320	3.28
Br12	26247949	26407230	4.54
Br13	29140520	29278562	6
Br14	30234740	30657006	6
Br15	37393634	37520213	6
Br16	37597617	37792182	3.14
Br17	42975813	43191580	6
Br18	43627794	44949482	6
Br19	55262853	55504204	3.34
Br20	65261344	65599705	2
Br21	65766782	66539510	6
Br22	86718101	86940017	6
Br23	87623079	87777799	3.58
Br24	92436593	92615949	4.55
Br25	93271624	93413960	3.48
Br26	94026338	94207976	4.58
Br27	97686171	98316954	6
Br28	98324295	100556043	6
Br29	100606043	101656560	6
Br30	101675083	103146801	6
Br31	104520695	107035981	6
Br32	107150652	107745702	6
Br33	109309294	109483402	3.34

WBS cells

	start coord	end coord	Value
Brick W1	388239	582463	4.06
Brick W2	851478	1566580	6
Brick W3	1590621	2549396	6
Brick W4	3556562	3775428	3.75
Brick W5	4904586	6673967	6
Brick W6	7747475	8124795	6
Brick W7	16476525	16642433	3.24
Brick W8	20437663	20582553	4.47
Brick W9	22088651	22214029	3.05
Brick W10	23400569	23609084	3.23
Brick W11	24740039	25024153	5.59
Brick W12	25089329	25266447	5.18
Brick W13	26221138	26552308	5.32
Brick W14	35039552	35242515	3.87
Brick W15	36957886	37087153	4.43
Brick W16	39584054	39769287	4.57
Brick W17	43845573	44371707	6
Brick W18	44431636	44683380	6
Brick W19	44695920	45245316	6
Brick W20	47654447	47875060	5.1
Brick W21	55394036	55666128	4.31
Brick W22	55979844	56247751	1.25
Brick W23	65422957	65745307	2.74
Brick W24	65885907	66570188	6
Brick W25	72301356	72576255	3.96
Brick W26	90203516	90378516	5.96
Brick W27	92143195	92380849	4.16
Brick W28	92455822	92632403	3.02
Brick W29	97704153	97957482	6
Brick W30	98254751	100556043	6
Brick W31	100606043	103117656	6
Brick W32	103292169	103679070	6
Brick W33	104209442	104432063	5.24

37267913	6
37402907	-4.35
37900394	6
38098772	-3.73
39124453	-6
39623268	-3.83
40539940	-6
42024114	5.61
47470045	-3.36
47715332	3.05
54640276	-6
55474127	-4.7
56042729	-1.26
69681084	-1.48
71943928	-3.25
77109252	1.94
77441106	-1.65
77615483	1.51
78498131	-6
90339628	3.25
91757093	-3.41
92130064	-3.01
93131618	-3.18
94014747	-6
98439624	-5.91
99383111	4.46
100556043	-4.44
100765921	-4.44
100959341	3.98
103207693	-3.44
103426082	-3.87
105422826	6
105690196	-5.94
106058937	-5.6
106640661	3.11
107892930	-6
110709877	-5.99
111848100	-6
112086718	-3.1
112919057	-5.82

Br34	110484427	110613145	5.6
Br35	116130224	116221175	5.48
Br36	116518419	116941001	5.7
Br37	127498590	127741534	6
Br38	127753054	130154523	6
Br39	130254523	130300381	6
Br40	130432206	130610290	6
Br41	130648382	131442288	6
Br42	131807636	131909634	3.63
Br43	132038708	132180324	5.8
Br44	132486653	132966837	6
Br45	133048439	133176750	3.19
Br46	133214331	133368615	5.83
Br47	133965913	134118502	5.26
Br48	134365715	134512321	6
Br49	134518926	134667337	6
Br50	135027863	135375113	5.12
Br51	135487478	135655086	6
Br52	136610210	136730764	3.51
Br53	138175659	138424568	4.12
Br54	138619468	139379377	6
Br55	139404377	140447383	6
Br56	140453277	140919562	6
Br57	141538782	141735524	4.23
Br58	141943082	142048195	5.43
Br59	142098195	142133054	5.43
Br60	148268281	149590100	6
Br61	149676798	152727248	6
Br62	153304880	153708586	5.76
Br63	155180210	155672264	6
Br64	155710464	155929674	6
Br65	156644513	156925170	5.81
Br66	157004889	157457833	6

Brick W34	104525837	105477558	6
Brick W35	105779271	106267463	5.78
Brick W36	106300808	106689583	5.9
Brick W37	106804274	107050631	5.02
Brick W38	107191057	107423410	3.35
Brick W39	107437119	107794363	5.58
Brick W40	111497753	111835409	5.87
Brick W41	112668300	112817117	3.41
Brick W42	116381530	116841517	5.69
Brick W43	117302637	117429507	4.3
Brick W44	119865913	120003887	4.43
Brick W45	120195847	120312715	4.3
Brick W46	120505944	120749320	6
Brick W47	121594985	121730174	4.44
Brick W48	126185048	126367509	3.69
Brick W49	127498590	127741534	6
Brick W50	127809263	128383487	6
Brick W51	128485564	129083947	6
Brick W52	129110056	130004914	6
Brick W53	130084975	130154523	6
Brick W54	130254523	130930795	6
Brick W55	131055625	131449375	5.84
Brick W56	132625697	132807413	6
Brick W57	132998060	133310909	5.91
Brick W58	133360811	133449577	3.2
Brick W59	133743630	133955985	5.99
Brick W60	134180867	134352747	5.94
Brick W61	134483361	135457312	6
Brick W62	136759134	136986598	3.29
Brick W63	137804266	138385743	6
Brick W64	138413722	139379377	6
Brick W65	139404377	140868005	6
Brick W66	141047843	141370809	6
Brick W67	141393003	141550864	5.93
Brick W68	141784404	141970909	5.25
Brick W69	142146676	142276197	5.41
Brick W70	142326197	142376709	5.41
Brick W71	142648732	142786039	5.56
Brick W72	148283139	149695313	6
Brick W73	149804501	152768389	6

116410039	-6
118445491	6
120196645	6
120778391	3.28
121181602	6
121385347	-5.93
123686831	6
127259650	6
127973294	6
128645619	-3.88
129475300	-5.81
130036645	-4.91
130584437	3.7
130867968	3.34
131296780	3.86
131489152	-6
131904233	6
132200307	-6
132992832	-5.91
133419226	-3.36
134334664	-6
134799482	5.5
136044890	6
137558106	4.35
137945590	5.14
138572825	-4.36
139379377	6
139850300	6
140712793	3.03
141070871	-6
141631058	3.97
142002937	-5.35
142276197	5.17
142358028	5.17
142615971	-5.51
144480222	6
144917684	-6
148144545	-6
148420876	-6
149579844	-6

Brick W74	154916850	155658082	6
Brick W75	156201089	157880022	6

150870503	3.63
151684409	-5.48
152706057	3.01
154052593	6
154866021	5.24
155125615	-6
156370222	-5.97
157444349	4.26

KCTD7 VIEWPOINT

Ratio BRICKs

	start coord	end coord	Value
Brick R1	3110444	3510010	6
Brick R2	3556562	3832043	5.95
Brick R3	7417749	7827469	-6
Brick R4	16088735	16745972	6
Brick R5	23111595	23289395	4.56
Brick R6	23542107	23917603	3.5
Brick R7	24269248	24741592	-5.78
Brick R8	24900084	25024153	6
Brick R9	26395804	26552308	3.45
Brick R10	28956475	29269098	-6
Brick R11	30262769	30433021	-4.67
Brick R12	34552649	35224277	6
Brick R13	37313573	37868864	-6
Brick R14	43021782	43323792	-5.99
Brick R15	43797579	44043488	-6
Brick R16	47732494	47987030	3.02
Brick R17	55262852	55395960	-1.87
Brick R18	55516259	55666128	2.65
Brick R19	56046181	56306040	1.18
Brick R20	87623079	87777799	-3.39
Brick R21	93271623	93413960	-3.78
Brick R22	94006430	94141151	-4.92
Brick R23	94137772	94608714	6
Brick R24	97971285	98362443	-6
Brick R25	98640373	98841090	-3.19
Brick R26	103406958	103618607	4.74
Brick R27	104083975	104503045	6
Brick R28	105491313	105657314	-4.46
Brick R29	106653602	106837351	-4.47
Brick R30	109011180	109330293	6
Brick R31	109330293	109482448	-5.78
Brick R32	110471654	110613145	-5.88
Brick R33	111737457	111849081	5.81

Control cells

	start coord	end coord	Value
Brick 1	497363	739221	5.83
Brick 2	1510259	1785892	5.99
Brick 3	1921106	2191292	3.45
Brick 4	2743153	3021187	5.85
Brick 5	4744850	6013329	6
Brick 6	6055626	6534976	5.8
Brick 7	10995812	11170429	3.25
Brick 8	12482071	12647718	5.53
Brick 9	13483091	13652084	5.81
Brick 10	17341056	17922841	6
Brick 11	20047545	20492237	5.99
Brick 12	21185355	21751187	6
Brick 13	21926470	22109747	6
Brick 14	22463950	22622181	5.66
Brick 15	22978316	23492980	6
Brick 16	24713501	24865451	4.73
Brick 17	25660421	25734119	3.62
Brick 18	26132005	26644354	6
Brick 19	27323992	27474057	3.11
Brick 20	27619935	27807240	3.94
Brick 21	28316685	28478670	5.8
Brick 22	29825956	30268151	6
Brick 23	31357623	31482717	4.22
Brick 24	31688459	31918277	6
Brick 25	32266785	32471077	6
Brick 26	33708584	33889746	3.58
Brick 27	36124431	36366208	3.18
Brick 28	37488785	37714776	6
Brick 29	37763862	37933770	3.89
Brick 30	38883351	39045413	3.65
Brick 31	39584054	39769287	5.71
Brick 32	41826324	42073694	5.98
Brick 33	42423538	42560222	5.91

WBS cells

	start coord
Brick W1	15217
Brick W2	635661
Brick W3	1993890
Brick W4	4609282
Brick W5	5472906
Brick W6	7709027
Brick W7	8111978
Brick W8	8609369
Brick W9	9127280
Brick W10	10638193
Brick W11	12932619
Brick W12	13904283
Brick W13	14598435
Brick W14	17126608
Brick W15	17833616
Brick W16	19957578
Brick W17	22760599
Brick W18	23191379
Brick W19	24741233
Brick W20	25184966
Brick W21	28348425
Brick W22	31906025
Brick W23	32476742
Brick W24	32955495
Brick W25	34815889
Brick W26	35773288
Brick W27	36086690
Brick W28	36947164
Brick W29	38060715
Brick W30	39135245
Brick W31	39877566
Brick W32	41847978
Brick W33	42744004

Brick R34	116745688	116915999	-3.15
Brick R35	117180083	117345220	6
Brick R36	119604910	119931709	3.46
Brick R37	120064438	120389714	6
Brick R38	120540112	120690270	3.3
Brick R39	121635418	122131955	6
Brick R40	129031524	129172796	-4.46
Brick R41	130766258	131136319	-5.82
Brick R42	131601226	131955965	-6
Brick R43	132038708	132180324	-6
Brick R44	132517243	132657609	-3.48
Brick R45	132783899	132966837	-6
Brick R46	132985512	133130829	5.9
Brick R47	133277168	133408939	-4.01
Brick R48	133739949	133883582	3.41
Brick R49	134007591	134146454	-4.56
Brick R50	134397511	134528059	-4.17
Brick R51	135299427	135442082	6
Brick R52	135461996	135622095	-6
Brick R53	136469531	136786068	-6
Brick R54	136786068	136979774	4.49
Brick R55	137761447	137935449	3.23
Brick R56	139760637	139949882	-3.28
Brick R57	140384229	140544165	4.77
Brick R58	141214666	141411735	4
Brick R59	141443601	141807690	-6
Brick R60	141807690	141897660	4.49
Brick R61	142107056	142276197	6
Brick R62	142326197	142376709	6
Brick R63	148270055	148426023	-6
Brick R64	149200369	149437114	-4.16
Brick R65	150206688	150498383	-6
Brick R66	151318994	151501243	-5.92
Brick R67	151661568	151902248	-3.47
Brick R68	152733031	153055209	-4.6
Brick R69	153103024	153833751	-6
Brick R70	155582945	156007346	-5.58
Brick R71	157861357	158067602	-3.48

Brick 34	43201701	43763633	6
Brick 35	43806249	44311762	4.53
Brick 36	44388141	45326843	6
Brick 37	45608475	45825762	4.33
Brick 38	47107129	47254873	6
Brick 39	54983096	55125559	2.92
Brick 40	55459533	55655948	4.09
Brick 41	55913174	56101792	5.56
Brick 42	56713069	56946177	1.85
Brick 43	63584435	63815752	1.09
Brick 44	68830596	69104918	4.1
Brick 45	69280430	69385108	1.41
Brick 46	70144528	70261386	1.57
Brick 47	71722568	71939658	2.48
Brick 48	72003322	72524150	6
Brick 49	72619864	73152680	5.87
Brick 50	73392089	73776834	2.73
Brick 51	74136155	74562950	3.76
Brick 52	74640826	74715724	4.21
Brick 53	74765724	74912730	4.21
Brick 54	74944782	75472564	2.79
Brick 55	75579695	76270462	5.98
Brick 56	76934525	77325446	6
Brick 57	77470265	77647839	3.91
Brick 58	77723112	77845977	1.71
Brick 59	80710607	80955831	3.66
Brick 60	81430553	81669287	5.86
Brick 61	83738719	83910417	6
Brick 62	84103291	84247675	4.33
Brick 63	86308906	87386282	6
Brick 64	87518014	87719055	4.56
Brick 65	89697255	89866882	5.82
Brick 66	90203516	90538816	5.97
Brick 67	91457796	91891441	5.82
Brick 68	92142256	92373678	4.97
Brick 69	92390552	92489161	4.69
Brick 70	93383199	93587302	5.88
Brick 71	94532731	94616200	3.04
Brick 72	97870669	98063521	5.11
Brick 73	98324294	98564761	4.75

Brick W34	43645153
Brick W35	44923042
Brick W36	50116300
Brick W37	50473123
Brick W38	54172545
Brick W39	54785749
Brick W40	56713069
Brick W41	65196747
Brick W42	68604985
Brick W43	69051456
Brick W44	69306377
Brick W45	69784338
Brick W46	70136211
Brick W47	71906234
Brick W48	72644422
Brick W49	74155912
Brick W50	74584454
Brick W51	74765724
Brick W52	74854195
Brick W53	75453666
Brick W54	76595247
Brick W55	76820877
Brick W56	77282884
Brick W57	78088178
Brick W58	78559934
Brick W59	80741192
Brick W60	86591690
Brick W61	87214857
Brick W62	87958360
Brick W63	90852682
Brick W64	91347072
Brick W65	91674440
Brick W66	93897959
Brick W67	94070801
Brick W68	96723792
Brick W69	97126872
Brick W70	97558137
Brick W71	98858832
Brick W72	99348112
Brick W73	100141767

Brick 74	99168981	99366241	5.9	Brick W74	100418162
Brick 75	99461192	100141251	6	Brick W75	100606043
Brick 76	100192021	100409743	6	Brick W76	101333067
Brick 77	101840783	102450376	6	Brick W77	101829143
Brick 78	102574712	102825575	6	Brick W78	102490980
Brick 79	102847450	103006744	6	Brick W79	102938876
Brick 80	104060035	104529019	6	Brick W80	104050471
Brick 81	104866596	105153837	3.1	Brick W81	104693345
Brick 82	105636407	105858893	4.68	Brick W82	105629499
Brick 83	107590882	108555208	6	Brick W83	106653602
Brick 84	109078819	109411152	5.64	Brick W84	110017318
Brick 85	110475468	110565542	5.41	Brick W85	110817703
Brick 86	111384734	111827857	6	Brick W86	112417580
Brick 87	112851787	113007775	3.15	Brick W87	113560194
Brick 88	114493829	114660998	6	Brick W88	114185044
Brick 89	115027224	115168370	6	Brick W89	115809576
Brick 90	116334234	116891262	6	Brick W90	116325922
Brick 91	119841483	119985881	4.93	Brick W91	116482169
Brick 92	120525283	120967786	6	Brick W92	116930308
Brick 93	121042195	121284031	5.87	Brick W93	118019485
Brick 94	123111736	123320116	6	Brick W94	118709087
Brick 95	127913442	128859782	6	Brick W95	118901065
Brick 96	128970270	129088793	5.28	Brick W96	119743161
Brick 97	129430732	129785625	6	Brick W97	120958855
Brick 98	129820946	130154523	6	Brick W98	121663354
Brick 99	130254523	130557788	6	Brick W99	126499455
Brick 100	132469930	132606602	6	Brick W100	126743396
Brick 101	134327928	134466040	5.83	Brick W101	127519382
Brick 102	134650242	134924359	5.99	Brick W102	127985106
Brick 103	135045870	135436043	6	Brick W103	128542051
Brick 104	136730771	136905121	3.14	Brick W104	128972366
Brick 105	137523119	137646562	5.9	Brick W105	129498913
Brick 106	137746957	139379377	6	Brick W106	129969270
Brick 107	139404377	139529773	6	Brick W107	130129746
Brick 108	139577058	139755527	6	Brick W108	130254523
Brick 109	139894702	140516107	6	Brick W109	131296786
Brick 110	140712794	140862886	3.93	Brick W110	131686681
Brick 111	141312295	141515127	4.3	Brick W111	131988659
Brick 112	141985659	142048195	6	Brick W112	132229807
Brick 113	142098195	142276197	6	Brick W113	134002835

Brick 114	142326197	142377681	6
Brick 115	142475673	142611544	6
Brick 116	143002861	143134440	6
Brick 117	143891856	144063383	5.44
Brick 118	144136154	144273348	5.57
Brick 119	148026830	148392380	5.92
Brick 120	148654485	149284089	5.76
Brick 121	149301816	149459426	5.76
Brick 122	149879659	150485978	6
Brick 123	150843518	151376786	6
Brick 124	151501516	152732731	6
Brick 125	153680555	153833751	4.27
Brick 126	155037382	155260755	4.04
Brick 127	155503209	155659062	6
Brick 128	155920137	156118071	3.61
Brick 129	156318947	156538523	5.95
Brick 130	156558028	156853249	6
Brick 131	156900603	157065353	6
Brick 132	157249889	157459265	5.99
Brick 133	158035629	158707091	6
Brick 134	158945734	159125509	3.28

Brick W114	134797947
Brick W115	135015898
Brick W116	135601811
Brick W117	137437032
Brick W118	137804267
Brick W119	138521862
Brick W120	138774119
Brick W121	139354185
Brick W122	139404377
Brick W123	141612024
Brick W124	142221228
Brick W125	142326197
Brick W126	142626761
Brick W127	143119168
Brick W128	144570732
Brick W129	148126506
Brick W130	148604477
Brick W131	150317977
Brick W132	151113404
Brick W133	152450439
Brick W134	154510639
Brick W135	156120201
Brick W136	156941382
Brick W137	157848940

end coord	Value
86684	4.27
881291	5.58
3392068	6
5241769	6
7386300	6
8031313	5.96
8310412	4.89
8906427	5.95
9324475	3.63
10804713	4.89
13079210	6
14040998	4.81
14925897	6
17307746	5.62
17982124	6
20202229	5.95
22947057	6
24159056	6
25031515	5.94
25280597	3.44
28478670	5.78
32071195	4.19
32912110	6
33523073	6
35099374	5.92
35964581	6
36194222	3.85
37052563	3.79
38283711	6
39258478	4.33
40116100	3.52
41972793	3.32
42898320	5.91

Ratio BRICKs

	start coord	end coord	Value
Brick R1	15217	197207	5.96
Brick R2	431788	660944	-3.66
Brick R3	1197066	1566580	-3.06
Brick R4	3040619	3410924	3.79
Brick R5	5107546	5368883	-4
Brick R6	6673974	7019967	5.41
Brick R7	7280228	7444781	6
Brick R8	7624936	7761266	4.6
Brick R9	7798008	7966874	6
Brick R10	8184890	8385278	5.99
Brick R11	8569134	9036952	6
Brick R12	9037746	9327211	5.76
Brick R13	10821418	11069627	-3.08
Brick R14	12426160	12705951	-6
Brick R15	12783009	13134779	6
Brick R16	13510640	13896517	-6
Brick R17	13894840	14359536	6
Brick R18	14549896	15004326	6
Brick R19	17461964	17858933	-6
Brick R20	17939094	18131321	5.89
Brick R21	19721352	20189538	6
Brick R22	20202236	20492448	-3.8
Brick R23	21185355	22188812	-6
Brick R24	22406241	22694411	-5.9
Brick R25	22949849	23284270	-5.61
Brick R26	23502601	23542107	-3.88
Brick R27	23542107	24179845	6
Brick R28	24846043	24943169	3.85
Brick R29	25184966	25280597	4.07
Brick R30	25669395	25797425	-3.18
Brick R31	26110463	26766972	-6
Brick R32	27073888	27345210	5.85
Brick R33	27367051	27499051	-3.05

HIP1 VIEWPOINT

Control cells

	start coord	end coord	Value
Br1	15217	232484	6
Br2	282484	573584	6
Br3	879651	2157963	6
Br4	2406671	3363416	6
Br5	4324902	4939625	4.65
Br6	5239281	6687106	6
Br7	8285763	8436077	3.73
Br8	8609369	8716920	6
Br9	10176388	10328144	4.98
Br10	11143057	11362021	5.97
Br11	12384578	12705951	6
Br12	15461926	15576967	6
Br13	20265809	20585904	6
Br14	22386553	22705800	5.94
Br15	23103430	23806899	6
Br16	24295223	24468388	3
Br17	24579255	24759884	6
Br18	24888779	25196244	5.43
Br19	25636161	25729569	5.23
Br20	25888975	26117709	3.05
Br21	27487025	27786609	6
Br22	28660097	28772367	3.42
Br23	31398011	31611713	5.97
Br24	33621154	33866113	6
Br25	34120122	34277718	5.62
Br26	35706644	35864289	5.86
Br27	36170539	36438499	4.09
Br28	36727239	36902721	6
Br29	36913017	37016513	6
Br30	37380670	37755224	6
Br31	38123335	38650974	6
Br32	38824561	38976566	3.77
Br33	39394090	39506648	4.92

WBS cells

Brick W1
Brick W2
Brick W3
Brick W4
Brick W5
Brick W6
Brick W7
Brick W8
Brick W9
Brick W10
Brick W11
Brick W12
Brick W13
Brick W14
Brick W15
Brick W16
Brick W17
Brick W18
Brick W19
Brick W20
Brick W21
Brick W22
Brick W23
Brick W24
Brick W25
Brick W26
Brick W27
Brick W28
Brick W29
Brick W30
Brick W31
Brick W32
Brick W33

44109511	6	Brick R34	27537141	27677507	-4.55	Br34	43687916	44284271	6	Brick W34
45182582	6	Brick R35	28199017	28397928	6	Br35	44608473	45167344	6	Brick W35
50339481	3.11	Brick R36	28431206	28546417	-3.35	Br36	45489083	45752020	6	Brick W36
50614830	3.97	Brick R37	29607678	30221797	-6	Br37	45912320	46032458	4.24	Brick W37
54336426	2.89	Brick R38	31680369	31807011	-6	Br38	47647633	47862323	4.11	Brick W38
54985134	3.2	Brick R39	31936724	32109260	6	Br39	48955353	49469753	6	Brick W39
56948566	2.71	Brick R40	32099720	32396587	-6	Br40	49565246	49775113	4.68	Brick W40
65441168	1.01	Brick R41	32496364	32873421	6	Br41	50040054	50370631	6	Brick W41
68916764	1.05	Brick R42	33055613	33523073	6	Br42	50410631	50462524	6	Brick W42
69254721	2.88	Brick R43	33889074	34002516	-6	Br43	50547801	50737104	5.82	Brick W43
69438087	1.24	Brick R44	34943322	35170252	6	Br44	51218961	51681920	5.97	Brick W44
69915759	1.43	Brick R45	35619210	36001031	6	Br45	54705700	55004149	5.86	Brick W45
70261386	2.42	Brick R46	37041681	37158864	6	Br46	55124459	55279659	5.16	Brick W46
72637974	6	Brick R47	37485536	37933777	-6	Br47	55347292	55581196	5.09	Brick W47
72929511	3.29	Brick R48	37993401	38275402	5.11	Br48	56478580	56761427	5.95	Brick W48
74392202	1.7	Brick R49	38883351	39045413	-3.03	Br49	64442934	64945851	5.86	Brick W49
74715724	1.11	Brick R50	39038152	39301686	6	Br50	65535351	65780598	6	Brick W50
74803030	1.11	Brick R51	39556823	39709719	-4.93	Br51	65792860	66793543	6	Brick W51
75367972	5.41	Brick R52	39992763	40201912	6	Br52	66954533	67127576	3.55	Brick W52
76206993	6	Brick R53	41904127	42073694	-3.57	Br53	68380384	68675536	2.39	Brick W53
76727975	3.66	Brick R54	42385729	42744004	-5.82	Br54	69170340	69388594	2.95	Brick W54
77125579	5.81	Brick R55	42794881	42878800	3.73	Br55	69449887	69635943	1.67	Brick W55
77684487	6	Brick R56	43151044	43565783	-6	Br56	70171661	70424700	5.99	Brick W56
78266775	1.19	Brick R57	45326850	45695196	-3.5	Br57	71866776	72132875	1.07	Brick W57
78724459	4.99	Brick R58	47158443	47293904	-3.33	Br58	72183627	74715724	6	Brick W58
81033126	4.47	Brick R59	53487594	54177872	-5.82	Br59	74765724	75030898	6	Brick W59
86900444	5.63	Brick R60	54172545	54421130	5.66	Br60	75456878	76155020	2.82	Brick W60
87758764	5.85	Brick R61	54688033	54989911	6	Br61	76394719	76721889	1.75	Brick W61
88124753	6	Brick R62	54985141	55126886	-3.5	Br62	77252279	77476578	4.49	Brick W62
91012854	4.35	Brick R63	55431322	55633783	-1.2	Br63	84183965	84418297	6	Brick W63
91587987	6	Brick R64	55913174	56101792	-1.76	Br64	87621103	87776521	3.57	Brick W64
91891441	4.66	Brick R65	63498345	63788840	-3.11	Br65	87985352	88124753	5.96	Brick W65
94040699	3.16	Brick R66	68486731	68753209	1.37	Br66	88378767	88569455	3.18	Brick W66
94260434	6	Brick R67	68914050	69093695	-1.22	Br67	91468507	92001357	6	Brick W67
97038253	6	Brick R68	69767890	69915365	1.03	Br68	92171708	92397936	6	Brick W68
97252319	5.7	Brick R69	71334152	71829189	-2.03	Br69	93713680	93927205	4.55	Brick W69
98070541	6	Brick R70	71928536	72172080	1.34	Br70	96707467	97010534	6	Brick W70
99089842	6	Brick R71	73519904	73880714	-3.36	Br71	97527038	98021350	3.66	Brick W71
100115515	6	Brick R72	74392209	74562950	-1.28	Br72	98480658	98668546	3.03	Brick W72
100331947	3.84	Brick R73	75471409	75684278	2.67	Br73	99351487	100544151	6	Brick W73

100556043	5.97	Brick R74	76934525	77040693	1.28	Br74	100654643	101656560	6	Brick W74
100702150	5.97	Brick R75	77052335	77257256	-3.4	Br75	101691048	102433999	6	Brick W75
101705165	5.92	Brick R76	77288332	77518596	1.2	Br76	103117663	103359378	6	Brick W76
102102639	6	Brick R77	78364659	78966791	6	Br77	104864034	105384992	6	Brick W77
102825575	6	Brick R78	81346240	81751676	-6	Br78	105401788	105838621	6	Brick W78
103304302	6	Brick R79	83723635	83891296	-4.93	Br79	105903929	106410756	5.24	Brick W79
104221761	4.42	Brick R80	86347472	86500822	-4.19	Br80	106826200	107050185	6	Brick W80
105283374	6	Brick R81	86844145	87081492	-5.4	Br81	107155433	107406477	6	Brick W81
106502047	6	Brick R82	87292623	87525879	3.72	Br82	107423417	107590322	6	Brick W82
107050185	5.91	Brick R83	87838123	88205028	6	Br83	111419394	111540636	4.94	Brick W83
110174911	4.25	Brick R84	89250235	89802620	-6	Br84	114504813	114681527	6	Brick W84
110952559	3.47	Brick R85	90203425	90370698	-6	Br85	115754728	115871429	3.45	Brick W85
112776184	5.96	Brick R86	90938114	91055144	5.94	Br86	116062538	116181846	6	Brick W86
113782494	5.85	Brick R87	91178905	91414793	6	Br87	116220928	116354535	4.76	Brick W87
114513397	6	Brick R88	91619365	91791786	-3.45	Br88	116466595	116969079	6	Brick W88
115959957	6	Brick R89	92390551	92489161	-5.35	Br89	118388471	118626864	6	Brick W89
116471480	6	Brick R90	93286682	93506666	-6	Br90	119987157	120084010	3.59	Brick W90
116750885	6	Brick R91	93519619	93680173	-6	Br91	120459856	120740027	5.95	Brick W91
117125470	3.37	Brick R92	93897959	94040699	5.63	Br92	121560090	121646694	5.14	Brick W92
118142425	5.81	Brick R93	96665853	96949553	6	Br93	122125030	122262339	4.15	Brick W93
118827464	5.2	Brick R94	97098584	97252319	5.88	Br94	127448842	128204337	6	Brick W94
119225045	3.18	Brick R95	98853437	99069573	4.5	Br95	128240168	129391466	6	Brick W95
120247462	6	Brick R96	99378811	99670814	4.3	Br96	129494075	129654800	4.83	Brick W96
121384020	6	Brick R97	101202931	101683978	5.96	Br97	130277634	130555841	3.28	Brick W97
121897264	6	Brick R98	102825582	102998305	-5.95	Br98	130980284	131477479	6	Brick W98
126591139	3.17	Brick R99	103014261	103356903	5.98	Br99	132141711	132232750	3.18	Brick W99
126809399	5.95	Brick R100	103962298	104073801	4.77	Br100	133713697	133855204	4.34	Brick W100
127617495	4.38	Brick R101	104109010	104529019	-6	Br101	133908713	134888674	6	Brick W101
128248238	3.99	Brick R102	106011671	106511106	5.68	Br102	135540709	135686375	4.49	Brick W102
128689404	5.75	Brick R103	106752060	106886110	3.65	Br103	139264034	139379377	4.18	Brick W103
129104087	6	Brick R104	107795099	108024042	-5.01	Br104	139404377	139437919	4.18	Brick W104
129834124	6	Brick R105	108162434	108645025	-6	Br105	139640247	140277882	5.97	Brick W105
130094926	5.16	Brick R106	108969249	109471339	-6	Br106	141378640	141533023	4.48	Brick W106
130154523	6	Brick R107	110012352	110240921	6	Br107	141868444	142048195	6	Brick W107
130712207	6	Brick R108	110391644	110650424	-5.95	Br108	142098195	142276197	6	Brick W108
131488118	6	Brick R109	111332277	111893833	-6	Br109	142326197	142358028	6	Brick W109
131831677	6	Brick R110	112262720	112694365	5.76	Br110	143658079	143869204	6	Brick W110
132113020	3.79	Brick R111	112754420	112865665	-5.69	Br111	147827090	148022197	5.93	Brick W111
132417057	4.63	Brick R112	113456069	113820552	5.87	Br112	148355152	148523418	6	Brick W112
134365709	6	Brick R113	114109823	114222117	-5.19	Br113	149301816	149478740	4.73	Brick W113

134969123	6
135168892	4.74
135728821	5.97
137681746	5.88
138424568	6
138641293	5.88
139065699	6
139379377	5.79
139532431	5.79
141884588	5.94
142276197	3.87
142433120	3.87
143067601	6
143265132	3.66
144762504	5.88
148305160	5.93
149401230	6
150878431	6
152334254	6
152998566	6
155310617	6
156906512	6
157484753	6
158197911	5.91

Brick R114	114484236	114767058	-6
Brick R115	114853885	115029192	4.27
Brick R116	115060127	115256415	-5.87
Brick R117	115578144	115895038	6
Brick R118	116280400	116410039	-5.5
Brick R119	116486568	116931620	-6
Brick R120	116939378	117151805	3.49
Brick R121	118142158	118550255	-6
Brick R122	118574393	119148046	6
Brick R123	119661130	119767800	-4.08
Brick R124	119997123	120133250	6
Brick R125	120505944	120989438	-6
Brick R126	121001798	121130153	5.9
Brick R127	123024422	123253020	-6
Brick R128	123270488	123331131	3.54
Brick R129	126499455	126928211	5.99
Brick R130	127556300	127644526	4.18
Brick R131	128238160	128468126	-6
Brick R132	131978578	132107673	6
Brick R133	132286638	132446184	5.37
Brick R134	132446184	132717568	-6
Brick R135	134097325	134267175	5.14
Brick R136	134924366	135069534	5.49
Brick R137	135385594	135506112	4.68
Brick R138	135554070	135853176	6
Brick R139	136713072	136905121	-5.95
Brick R140	137450276	137620491	6
Brick R141	138036740	138272047	6
Brick R142	138327352	138563856	-5.91
Brick R143	138609126	139345588	-6
Brick R144	139546389	139897978	-5.92
Brick R145	140685188	140971738	-6
Brick R146	141100756	141329169	6
Brick R147	141380049	141529505	-5.74
Brick R148	141673075	141943076	6
Brick R149	142102816	142221221	-3.4
Brick R150	142464693	142574604	-3.32
Brick R151	142650838	143027066	6
Brick R152	143192341	143314979	3.61
Brick R153	143557313	143897296	6

Br114	149834088	150528428	6
Br115	150674509	150841875	4.07
Br116	150964690	151196000	3.7
Br117	151682918	151922328	6
Br118	154896658	155008637	4.44
Br119	155260762	155486958	5.48
Br120	155658089	155870366	3.97

Brick W114
Brick W115
Brick W116
Brick W117
Brick W118
Brick W119
Brick W120

Brick R154	144135277	144222112	-6
Brick R155	144459253	144797671	5.98
Brick R156	147696034	148475373	-6
Brick R157	149772975	149991592	-3.75
Brick R158	150329847	150852896	6
Brick R159	150853535	151225357	-4.42
Brick R160	151248549	151465527	4.91
Brick R161	152153864	152432821	-5.96
Brick R162	152495051	152893607	5.9
Brick R163	153531480	153708586	-5.97
Brick R164	154499601	154706516	6
Brick R165	154916850	155128594	3.37
Brick R166	155897199	156170844	-5.93
Brick R167	156430280	156579218	4.54
Brick R168	157004888	157376928	6
Brick R169	157756759	158325958	5.99
Brick R170	158301714	158563661	-6

start coord	end coord	Value
15217	170304	3.06
825191	1105390	3.95
1156447	3150004	6
3211566	3363416	3.81
4675690	5206014	6
5210601	6689109	6
7910809	8137550	6
8665355	8835205	6
11860561	12080611	5.33
12283564	12416965	4.34
12495661	12627828	4.18
18322918	18447170	5.97
20341571	20593430	5.93
21509228	21643855	4.39
22161550	22324989	3.7
23426238	23921163	6
24839845	24989900	6
24998468	25184959	6
25511158	25631617	6
26103157	26412439	5.87
28124837	28297391	3.69
28476113	28595269	6
28888776	29084445	5.78
29644652	29876224	6
30681794	30856567	3.16
31243955	31688584	6
31959272	32141789	5.81
36947164	37475775	6
37488785	37714776	4.69
38301984	38661518	6
39709726	39893927	3.04
43590905	44227713	6
44496999	45449314	6

Ratio BRICKs

	start coord	end coord	Value
Brick R1	34421	232484	-6
Brick R2	282484	520897	-6
Brick R3	2123427	2418354	6
Brick R4	2853354	3352301	-6
Brick R5	4305399	4768588	-5.92
Brick R6	6687113	6949138	-3.54
Brick R7	7960946	8117918	3.53
Brick R8	8222328	8622866	-6
Brick R9	8660257	8978700	6
Brick R10	10023245	10328144	-5.85
Brick R11	11156644	11245948	5.32
Brick R12	11272363	11687643	-6
Brick R13	11667867	12207503	6
Brick R14	12416972	12627828	-5.67
Brick R15	15131190	15636289	-6
Brick R16	18250867	18679015	6
Brick R17	20202236	20517046	-6
Brick R18	21476001	21643855	4.63
Brick R19	21980915	22378625	6
Brick R20	22415087	22721544	-6
Brick R21	23057421	23548438	-6
Brick R22	23542107	23909964	5.8
Brick R23	24160300	24531544	-5.93
Brick R24	24649307	24820937	-6
Brick R25	25024160	25216486	-5.86
Brick R26	25942953	26136937	-6
Brick R27	27203290	27534787	-5.98
Brick R28	27570285	27729337	-6
Brick R29	27718816	27874975	4.55
Brick R30	28036141	28604372	6
Brick R31	28604372	28805643	-5.88
Brick R32	28935991	29163573	5.91
Brick R33	29544150	29852590	6

GBAS VIEWPOINT
Control cells

	start coord	end coord	Value
Br1	536257	764025	3.18
Br2	876058	1244223	3.2
Br3	1566587	2105112	5.81
Br4	2406671	2659147	5.99
Br5	3269417	3507534	6
Br6	3976761	4171925	5.87
Br7	4334997	4573333	4.52
Br8	5206021	5668255	5.21
Br9	6172719	6472579	5.78
Br10	6654351	6993491	4.86
Br11	7229385	7410581	6
Br12	7794193	7964396	3.11
Br13	8022228	8247759	6
Br14	8443855	8518609	5.76
Br15	8783725	8936582	5.89
Br16	9683194	9845966	3.19
Br17	9933168	10564323	6
Br18	10990874	11164320	4.65
Br19	11273177	11452355	5.96
Br20	13786349	14017188	6
Br21	15435986	15546595	3.5
Br22	16344222	16529402	6
Br23	16831438	17341049	6
Br24	17508723	17864832	6
Br25	18457483	19130066	6
Br26	21643861	21926464	5.75
Br27	22044895	22189081	3.81
Br28	22368948	22866973	6
Br29	22882419	23016754	5.77
Br30	24125286	24306411	6
Br31	24522693	25187005	6
Br32	25199661	25337315	5.59
Br33	25614068	25734119	6

45692132	45910503	6	Brick R34	31168681	31334281	6	Br34	26265315	26674199	5.89
50542242	50724099	3.2	Brick R35	31531061	31722643	6	Br35	26935838	27134240	5.98
51304875	51525618	3.58	Brick R36	31751473	32257763	6	Br36	28293578	28470531	3.76
51679338	51893536	6	Brick R37	33517713	33918735	-6	Br37	28647659	28772367	5.81
52579511	52733548	5.76	Brick R38	34076290	34339036	-5.9	Br38	29330849	29507029	6
54495595	54697175	1.87	Brick R39	35773288	35964581	-5.15	Br39	31013992	31169019	6
54715133	54938700	6	Brick R40	36162628	36720987	-5.96	Br40	32506320	32726156	3.73
55090099	55209739	2.23	Brick R41	36894649	36949457	-6	Br41	32907187	33108339	4.4
55385022	55971717	6	Brick R42	36949457	37404012	6	Br42	33280445	33717838	5.7
56279892	56462811	3.59	Brick R43	37458587	37599638	-5.45	Br43	35688423	35838517	5.99
65185368	65672844	3.23	Brick R44	37599644	37967866	6	Br44	36124431	36366208	5.99
65792859	66855212	5.99	Brick R45	38016577	38522205	-6	Br45	37717113	38145431	5.91
66917332	67127576	4.44	Brick R46	38549207	38722382	5.95	Br46	38275409	38452278	6
67707542	67881805	1.18	Brick R47	38813781	38976566	-5.17	Br47	39458377	39533571	4.01
68576430	68792505	1.15	Brick R48	39373730	39533208	-5.89	Br48	39648140	39795921	4.42
69015232	69639426	5.82	Brick R49	39702139	39889522	5.92	Br49	43315561	43418781	3.57
70138978	70263243	6	Brick R50	43529150	43645146	-5.34	Br50	43645153	43835208	5.66
71928535	72174107	2.13	Brick R51	43645153	43827764	4.84	Br51	43965627	44213424	4.34
72177747	72927722	6	Brick R52	44990623	45272599	6	Br52	45121818	45575605	6
73152475	74554803	6	Brick R53	45637902	45735243	-4.31	Br53	47181943	47313197	3.85
75936032	76141489	2.5	Brick R54	45786775	45910503	6	Br54	47709672	47942457	6
76394720	76576152	1.67	Brick R55	45901229	46150876	-5.94	Br55	51187683	51394051	4.01
77398681	77596251	1.44	Brick R56	47590705	47692724	3.28	Br56	55255089	55459526	1.72
84196637	84431143	3.25	Brick R57	47771836	48006659	-6	Br57	62889656	63156441	5.32
86292445	86425384	4.97	Brick R58	48329908	49865081	-6	Br58	63957196	64590836	6
87672448	87823823	5.9	Brick R59	50175732	50370631	-4.48	Br59	64934889	65164218	2.39
87985352	88124753	3.52	Brick R60	50410631	50420747	-4.48	Br60	65169252	65772166	5.83
88554705	88673490	3.16	Brick R61	51737572	52129103	6	Br61	66311296	66516658	1.47
91097012	91306811	6	Brick R62	52604057	52802113	5.82	Br62	69094156	69364800	1.25
91474449	91702070	5.97	Brick R63	54487625	54715133	6	Br63	69456309	69609155	4.12
92015261	92576744	6	Brick R64	54751623	54938700	-1.83	Br64	70086567	70160458	3.57
95416815	95515305	5.44	Brick R65	55090099	55125559	3	Br65	70462506	70670068	2.31
97604154	97891811	3.78	Brick R66	55144868	55289569	-4.01	Br66	71001243	71250631	5.34
98285549	99171728	6	Brick R67	55536527	55965522	6	Br67	71352149	72132875	5.33
99400716	100202916	6	Brick R68	55979844	56454754	5.77	Br68	72170820	72279697	2.62
100692075	101658502	6	Brick R69	56553694	56839629	-6	Br69	73591184	73841286	3.25
101700704	102447182	6	Brick R70	64306277	65185361	-6	Br70	75727472	76153698	6
103143680	103301625	6	Brick R71	65205701	65448087	1.1	Br71	76699810	76833276	1.26
103359385	103599069	4.32	Brick R72	65512441	65747119	-5.37	Br72	78211699	78423214	6
104000645	104134910	3.78	Brick R73	66471211	66531322	1.18	Br73	78749375	78911377	5

104881158	105212591	6	Brick R74	66773776	66990932	2.68	Br74	80235585	80396076	5.1
105306594	105921797	6	Brick R75	68260550	68675536	-5.88	Br75	80705288	80987970	6
107315868	107675365	6	Brick R76	68675542	68792505	2.77	Br76	81140640	81328869	6
111702953	111836259	5.99	Brick R77	69015232	69175904	4.16	Br77	82105483	82294610	5.9
114552010	114800516	5.91	Brick R78	69364807	69527934	4.27	Br78	83240150	83429596	6
116058866	116177816	5.83	Brick R79	69633219	69806787	-2.37	Br79	83533803	83766917	6
116516732	116939371	5.58	Brick R80	70206856	70526284	-1.11	Br80	87066277	87326059	6
117278281	117424687	5.55	Brick R81	72434007	72927722	-1.51	Br81	87623079	87777799	5.33
117622506	117777498	4.28	Brick R82	73031414	73701392	-3.43	Br82	87925691	88006931	5.65
120507804	120643113	4.17	Brick R83	74039619	74715724	-1.97	Br83	88672160	88826960	6
124520775	124659556	5.37	Brick R84	74765724	75165373	-1.97	Br84	89125213	89318191	3.53
127436567	128396698	6	Brick R85	75456878	75718777	-1.22	Br85	90651965	90740066	5.07
128444804	128973132	6	Brick R86	76564645	76702104	-1.1	Br86	92133046	92310802	4.44
129913625	130045462	3.56	Brick R87	77244333	77470258	-3.05	Br87	94550779	94892473	6
130370493	130758202	5.99	Brick R88	84167946	84375081	-3.6	Br88	95588537	96499530	6
130769240	130983361	5.51	Brick R89	86238430	86451411	6	Br89	97509655	97825612	3.01
131052990	131481017	6	Brick R90	88259957	88569462	-5.94	Br90	101992531	102579394	6
131625539	131719948	5.19	Brick R91	88620293	88749358	5.72	Br91	102809945	102991326	6
131813922	131899379	4.39	Brick R92	91761821	92001357	-6	Br92	103634293	103958725	6
133637092	133938153	6	Brick R93	92398259	92504885	5.74	Br93	104062186	104265219	3.96
133999720	134348227	6	Brick R94	93587309	93847959	-4.51	Br94	104495000	104614335	5.95
134380404	134892623	6	Brick R95	93923228	94040699	-3.04	Br95	106866873	107406477	6
135639365	135810251	6	Brick R96	95443932	95597865	6	Br96	107677075	108062363	5.99
136905128	137088108	6	Brick R97	96486930	97380553	-6	Br97	110852200	110961490	5.94
137732491	138177492	5.76	Brick R98	98173657	98373002	-5.92	Br98	111586387	111794677	4.68
139247068	139379377	5.88	Brick R99	98645409	99089842	6	Br99	112361774	112572864	4.87
139404377	139590778	5.88	Brick R100	99392429	99720243	-3.02	Br100	114233997	114447769	6
139844668	140115229	4.78	Brick R101	100028990	100469014	-5.84	Br101	115080526	115271816	5.79
140497017	140703508	6	Brick R102	103378904	103608313	5.89	Br102	116081844	116205027	6
140712794	140885720	5.92	Brick R103	103821247	104188403	6	Br103	117447182	117576089	4.64
141078475	141181151	5.33	Brick R104	105763071	105903929	5.82	Br104	117788042	117972757	6
141667990	141853629	4.34	Brick R105	105903929	106274232	-5.94	Br105	117992531	118119463	4.19
142122880	142262884	5.44	Brick R106	106285894	106525386	-6	Br106	120302195	120446621	3.59
142692959	142927261	6	Brick R107	106847759	107050185	-4.23	Br107	120749327	120875511	4.06
143715018	143865982	4.6	Brick R108	107285509	107486107	-6	Br108	120920035	121245328	5.85
145763515	145855001	4.47	Brick R109	107608508	107794363	3.7	Br109	121866483	122004868	3.57
148131134	149341648	6	Brick R110	111409930	111660218	-5.83	Br110	122077898	122249174	6
149675782	149874146	3.07	Brick R111	111740350	111901098	6	Br111	123323120	123398083	3.23
149881002	150496898	5.95	Brick R112	114689749	114917930	3.77	Br112	123827930	123924840	4.35
150849451	151529013	6	Brick R113	115664468	115847975	-6	Br113	124265883	124547066	6

151896528	152334254	6
153479360	154038540	6
154559144	154778879	6
155125622	155310617	5.02
155311362	155521867	4.98
155869973	156255540	6
156266883	157070205	6

Brick R114	116237221	116381053	-3.83
Brick R115	116426912	116699628	-3.15
Brick R116	116754210	116931620	-3.09
Brick R117	117550448	117712265	4.6
Brick R118	117742197	117924292	3.64
Brick R119	118137797	119839474	-6
Brick R120	119987157	120084010	-3.37
Brick R121	120311709	120507797	3.17
Brick R122	120557127	120849069	-5.97
Brick R123	121560083	121646694	-5.94
Brick R124	122125030	122262339	-3.41
Brick R125	124359504	124654325	6
Brick R126	127397642	127519375	3.65
Brick R127	127906222	128061993	-5.83
Brick R128	128319854	128529188	-6
Brick R129	128521819	128599252	3.69
Brick R130	128687471	129329843	-6
Brick R131	129502791	129692375	-4.3
Brick R132	130017079	130154523	3.91
Brick R133	130524768	130719612	6
Brick R134	131099050	131295025	-4.97
Brick R135	131452389	131625532	-3.08
Brick R136	131813922	131953494	4.49
Brick R137	132084694	132219020	-6
Brick R138	133833998	133910512	4.55
Brick R139	133955101	134104842	-6
Brick R140	134228782	134515142	-5.99
Brick R141	134851380	135023504	-3.57
Brick R142	135602138	136165339	5.93
Brick R143	136639210	137068304	6
Brick R144	137620498	138521855	6
Brick R145	139592022	139846755	-5.71
Brick R146	140447633	140862886	6
Brick R147	141017891	141281903	6
Brick R148	141432033	141709400	-6
Brick R149	141732962	141884595	5.76
Brick R150	141884595	142048195	-6
Brick R151	142098195	142245156	-6
Brick R152	142661784	143203547	6
Brick R153	143509575	143670892	4.7

Br114	126135021	126239101	3.52
Br115	126412905	126575642	4.73
Br116	127363482	127477380	5.77
Br117	127629775	127769427	6
Br118	127914660	128060787	3
Br119	133410917	133560245	3.42
Br120	135056655	135243149	3.52
Br121	135700906	135868404	6
Br122	136451397	136639203	4.45
Br123	138260733	138521855	6
Br124	139151243	139379377	6
Br125	139404377	139546383	6
Br126	140445661	140586248	6
Br127	140684731	141032916	6
Br128	141158196	141442158	5.98
Br129	142122880	142276197	6
Br130	142326197	142513231	6
Br131	143206919	143347897	6
Br132	143397897	144218881	6
Br133	146467497	146623014	5.88
Br134	147712961	148046310	5.79
Br135	148349533	148518996	6
Br136	148895549	149478740	6
Br137	149881002	150317152	6
Br138	150358008	150500140	3.42
Br139	150999048	151171613	3.19
Br140	151443415	151873907	5.87
Br141	151921133	152120913	6
Br142	153422171	153626052	6
Br143	154666209	154879603	4.33
Br144	155945372	156214442	6
Br145	156229231	156871687	6
Br146	156878331	157045222	3.95
Br147	158213814	158609501	5.94

Brick R154	143865576	144005108	3.12
Brick R155	145763515	145869716	5.8
Brick R156	147844117	148020063	-6
Brick R157	148132195	148397017	5.83
Brick R158	148498268	149301809	6
Brick R159	149301809	149443125	-5.96
Brick R160	150006894	150329847	-3.43
Brick R161	151103608	151470454	6
Brick R162	151682918	151896528	-6
Brick R163	151896528	152426663	6
Brick R164	153333656	154089623	6
Brick R165	154591417	154801843	4.2
Brick R166	154896658	155008637	-4.82
Brick R167	155807737	156110631	6
Brick R168	156953461	157154869	3.32

WBS cells

	start coord	end coord	Value
Brick W1	176003	232484	5.87
Brick W2	282484	702614	5.87
Brick W3	2464258	3083893	5.99
Brick W4	3215381	3393686	3.98
Brick W5	4209256	5363777	6
Brick W6	5404460	5630733	3.76
Brick W7	6235053	6770262	6
Brick W8	7640756	7985574	6
Brick W9	8184379	8504227	6
Brick W10	8738990	8897349	5.63
Brick W11	11272362	11450069	6
Brick W12	12284233	12443909	4.01
Brick W13	12580798	12866651	6
Brick W14	14549896	14757991	5.72
Brick W15	15705266	16088735	5.86
Brick W16	17105486	17272575	6
Brick W17	18449503	18749763	6
Brick W18	20382725	20529348	3.37
Brick W19	20781217	20900757	3.15
Brick W20	22615512	22988651	5.84
Brick W21	23958907	24468170	6
Brick W22	24943176	25064661	5.94
Brick W23	25260318	25388761	6
Brick W24	26331121	26497595	6
Brick W25	29759590	29919873	6
Brick W26	29985322	30430869	6
Brick W27	31225332	31477246	5.92
Brick W28	32458620	32685615	6
Brick W29	32908074	33302258	5.58
Brick W30	33485740	33966522	5.99
Brick W31	37135097	37485529	6
Brick W32	37877727	38043536	3.92
Brick W33	38811229	39011242	6

Ratio BRICKs

	start coord	end coord	Value
Brick R1	209718	232484	5.25
Brick R2	282484	541249	5.25
Brick R3	1545501	2105112	-6
Brick R4	2321837	2608067	-6
Brick R5	2878654	3352301	5.98
Brick R6	3410283	3621658	5.64
Brick R7	3726641	4301723	-6
Brick R8	4304321	4925190	4.84
Brick R9	6061221	6358994	-5.83
Brick R10	7488579	7681789	-3.58
Brick R11	7686078	7884991	6
Brick R12	8222328	8471480	6
Brick R13	9384989	9708326	6
Brick R14	9708326	9845966	-5
Brick R15	9908290	10363411	-6
Brick R16	12109899	12360903	5.76
Brick R17	12669608	12810857	4.05
Brick R18	13819150	13971784	-5.48
Brick R19	14584449	14737829	6
Brick R20	15633919	16076727	6
Brick R21	16850171	17008618	-3.05
Brick R22	17495381	17984253	-6
Brick R23	18341559	18493765	-3.58
Brick R24	18708539	19130066	-5.86
Brick R25	20781217	20944319	3.69
Brick R26	21630722	21945326	-5.99
Brick R27	22318608	22386553	3.03
Brick R28	22386553	22674873	-5.91
Brick R29	22748365	22921190	4.21
Brick R30	23959697	24130179	3.53
Brick R31	24159063	24377274	5.98
Brick R32	24699909	25005966	-6
Brick R33	25566877	25828455	-6

ZNF107 VIEWPOINT
Control cells

	start coord	end coord
Br1	1379457	1961348
Br2	2608094	2925858
Br3	3363422	3502751
Br4	4653116	5167268
Br5	6205903	6756865
Br6	8109670	8502788
Br7	8995902	9136344
Br8	10912935	11295011
Br9	14549895	14676088
Br10	15128565	15258856
Br11	15428003	15518995
Br12	20574860	20692298
Br13	21738949	21910358
Br14	22189087	22343728
Br15	22377435	22694411
Br16	23497898	23641775
Br17	24594245	24813187
Br18	24909796	25062502
Br19	25115212	25278003
Br20	25398457	25570831
Br21	25582001	25695996
Br22	28480954	28772367
Br23	28802552	29030567
Br24	29195549	29302111
Br25	31711444	31803460
Br26	31959271	32141789
Br27	32903679	33078170
Br28	33591914	33932101
Br29	37108915	37418276
Br30	37920400	38325260
Br31	38695511	38824554
Br32	40188890	40337184
Br33	41014914	41178454

Brick W34	39393442	39676481	5.8	Brick R34	26457621	26666971	-4.54	Br34	43015590	43201695
Brick W35	39864512	40101059	6	Brick R35	26760391	27148153	-6	Br35	43333276	43943537
Brick W36	40361519	40508425	3.54	Brick R36	28293578	28470531	-5.75	Br36	47266097	47662653
Brick W37	40719407	40895233	5.74	Brick R37	28671424	29131348	-5.81	Br37	47822932	48017887
Brick W38	41469238	41584502	4.76	Brick R38	29281579	29616134	-5.88	Br38	48316280	48504629
Brick W39	42236856	42385722	4.96	Brick R39	29884520	29999363	-4.59	Br39	50497000	50911786
Brick W40	42898327	43161268	6	Brick R40	30262770	30433021	5.93	Br40	51394558	51664801
Brick W41	43177482	43333919	4.67	Brick R41	31006918	31298927	-6	Br41	51827023	52022202
Brick W42	44414717	45175488	6	Brick R42	31298927	31459584	6	Br42	52804893	53009564
Brick W43	47561906	47787607	4.04	Brick R43	33272770	33592737	-5.86	Br43	53177563	53341570
Brick W44	48065412	48318011	5.42	Brick R44	33767226	33941238	4.31	Br44	54788956	55189785
Brick W45	48504636	48689037	3.94	Brick R45	36087900	36266631	-4.36	Br45	55289575	56344838
Brick W46	49816988	50020320	5.9	Brick R46	37145232	37485015	5.62	Br46	56369252	57308289
Brick W47	50254847	50370631	4.76	Brick R47	39294509	39418234	3.16	Br47	57312077	57573670
Brick W48	50410631	50458949	4.76	Brick R48	39503482	39676107	3.38	Br48	62461171	63247917
Brick W49	50632426	50809061	5.92	Brick R49	39799417	40034633	3.27	Br49	64714028	64979901
Brick W50	55538413	55724559	2.02	Brick R50	40371808	40528568	3.07	Br50	65098188	65635417
Brick W51	62501128	62750257	4.79	Brick R51	40719407	40895233	5.09	Br51	65982771	66257690
Brick W52	63887904	64235808	1.06	Brick R52	41362535	41568808	6	Br52	66415997	66548368
Brick W53	64306277	64809843	3.08	Brick R53	42146042	42259228	-6	Br53	67614036	67821154
Brick W54	65155639	66053234	6	Brick R54	43138429	43315554	3.83	Br54	67856289	68007351
Brick W55	66568168	66782620	1.71	Brick R55	44579770	45163234	6	Br55	68792511	69044297
Brick W56	67259100	67371518	1.19	Brick R56	45159082	45489076	-5.93	Br56	69778141	69915759
Brick W57	68070100	69426206	6	Brick R57	47208963	47369577	-6	Br57	70055363	70183703
Brick W58	69805401	70173830	6	Brick R58	47787614	47997129	-6	Br58	70810949	71001575
Brick W59	70894087	71568464	5.75	Brick R59	48466127	48756608	6	Br59	71237680	71375037
Brick W60	71661171	71913558	2.29	Brick R60	49787030	49970600	6	Br60	72162361	72339433
Brick W61	71958670	72172080	2.66	Brick R61	51188116	51394552	-6	Br61	73011191	73323144
Brick W62	72301356	72576255	3.79	Brick R62	55279665	55535203	-1.23	Br62	75309234	76109735
Brick W63	72599607	72861068	4.58	Brick R63	62496976	62716787	1.24	Br63	76543838	76644853
Brick W64	73127275	73323144	3.63	Brick R64	62919556	63166249	-2.44	Br64	76721267	76907698
Brick W65	73420494	73686975	4.85	Brick R65	64139376	64426613	-1.73	Br65	77052335	77186773
Brick W66	74912737	75165373	5.15	Brick R66	64817566	65039389	-1.79	Br66	77488509	77651946
Brick W67	75273392	75533294	2.92	Brick R67	65731245	66017052	5.28	Br67	77858111	78082480
Brick W68	75993489	76141489	1.81	Brick R68	66568168	66782620	1.56	Br68	79890403	80059545
Brick W69	76400794	76725419	5.54	Brick R69	67259100	67380113	4.51	Br69	80987976	81202647
Brick W70	76941974	77244326	5.92	Brick R70	68021084	68489103	4.63	Br70	81623668	81762150
Brick W71	77362188	77776548	6	Brick R71	68607082	69098843	5.9	Br71	81841555	82017453
Brick W72	77779197	77934125	5.73	Brick R72	69170340	69246024	1.02	Br72	83149485	83320408
Brick W73	78418290	78570494	4.53	Brick R73	69246031	69306370	-1.37	Br73	83910423	84103285

Brick W74	79470713	79622233	5.88	Brick R74	69306377	69434481	1.72	Br74	84363157	84706521
Brick W75	80091926	80307150	4.15	Brick R75	69434488	69609155	-2.98	Br75	86367243	86683154
Brick W76	81418653	81687938	5.89	Brick R76	69660214	70090042	5.89	Br76	86710339	87145237
Brick W77	83180900	83488527	5.68	Brick R77	70515571	70748710	-3.39	Br77	87632494	87805565
Brick W78	83547353	83719322	6	Brick R78	71250638	71425701	1.27	Br78	87996093	88156925
Brick W79	84196637	84431143	5.86	Brick R79	71518577	71748903	-2.69	Br79	88472664	88634495
Brick W80	84497162	84680570	5.57	Brick R80	71909981	71928529	1.31	Br80	89754427	90146454
Brick W81	86112469	86337549	4.48	Brick R81	72172087	72279697	-1.5	Br81	90318895	90673019
Brick W82	86680744	87081492	6	Brick R82	72301356	72756460	2.06	Br82	91230563	91571880
Brick W83	87976460	88333819	5.96	Brick R83	73127275	73323144	1.28	Br83	92348172	92574921
Brick W84	89893889	90072009	4.35	Brick R84	73420494	73591177	1.54	Br84	92819764	93052545
Brick W85	90137235	90362260	6	Brick R85	73686982	74033903	-1.92	Br85	94204470	94297921
Brick W86	90703648	90779406	4.56	Brick R86	74765724	75166972	2.89	Br86	94673519	95123136
Brick W87	92081881	92356336	6	Brick R87	75718777	76121914	-5.96	Br87	95847693	96257150
Brick W88	92939768	93104317	6	Brick R88	76141496	76317924	-1.4	Br88	96416125	96564267
Brick W89	93163061	93327793	5.89	Brick R89	76317924	76695369	6	Br89	98218895	98436305
Brick W90	93666247	93871295	5.99	Brick R90	76833283	77172745	3.23	Br90	98906037	99120868
Brick W91	94669334	94838805	3.91	Brick R91	78424615	78580825	6	Br91	99890759	100139537
Brick W92	94920058	95117779	6	Brick R92	78750992	78935236	-6	Br92	101418676	101627008
Brick W93	100826128	101638191	6	Brick R93	80624522	81009078	-6	Br93	102485949	102723466
Brick W94	101773119	101979240	5.3	Brick R94	81067402	81257066	-6	Br94	102863888	102979820
Brick W95	102890634	103153945	5.91	Brick R95	81413285	81656785	4.02	Br95	103181386	103313794
Brick W96	103356910	103509818	3.3	Brick R96	82017460	82498980	-6	Br96	104309903	104449722
Brick W97	103757711	103964360	4.04	Brick R97	83474039	83631399	3.15	Br97	104562822	105436867
Brick W98	104617586	104864327	3.05	Brick R98	83631405	83772722	-6	Br98	107795098	108096518
Brick W99	105325086	105482393	6	Brick R99	84418304	84761726	5.98	Br99	108112165	108328720
Brick W100	106803723	107050631	6	Brick R100	86153210	86359996	6	Br100	109222629	109428911
Brick W101	107677075	107811369	3.72	Brick R101	86634288	87072756	6	Br101	109520581	109814794
Brick W102	109200132	109411152	5.43	Brick R102	87228280	87492304	-6	Br102	110804031	110883040
Brick W103	110709884	110833773	6	Brick R103	87530187	87821377	-5.91	Br103	111509711	111712825
Brick W104	111586388	111794677	4.64	Brick R104	87958360	88287070	6	Br104	112872189	113062009
Brick W105	112099105	112504559	6	Brick R105	88670550	88799394	-5.88	Br105	113742113	113933605
Brick W106	113202780	113430858	3.08	Brick R106	89125213	89442138	-3.67	Br106	114767064	114949269
Brick W107	113886455	114116879	6	Brick R107	89912339	90072009	5.47	Br107	116039614	116164325
Brick W108	115720265	115979318	5.96	Brick R108	90069106	90146454	-5.66	Br108	117801799	117927301
Brick W109	117306941	117445405	3.56	Brick R109	90146461	90277884	4.09	Br109	119502141	119973948
Brick W110	117687340	117966261	6	Brick R110	90740072	90846475	4.72	Br110	122928424	123344857
Brick W111	119985888	120127238	6	Brick R111	91997549	92211674	3.62	Br111	123495620	123687916
Brick W112	120640763	120750054	5.63	Brick R112	92952033	93107978	5.36	Br112	126488235	126618482
Brick W113	121663354	121895169	6	Brick R113	93163061	93407165	6	Br113	127629774	127745270

Brick W114	123379092	123634553	6
Brick W115	125333763	125547439	6
Brick W116	126201044	126374505	4.95
Brick W117	127975519	128239475	3.15
Brick W118	128287845	128658192	6
Brick W119	132154758	132266778	5.21
Brick W120	134698255	134851373	5.72
Brick W121	134950580	135408791	6
Brick W122	135472550	135649897	6
Brick W123	137224594	137358122	4.61
Brick W124	138413722	139219254	6
Brick W125	139327606	139379377	5.64
Brick W126	139404377	139502270	5.64
Brick W127	141150036	141305259	5.93
Brick W128	141381422	141784397	6
Brick W129	141868444	141996524	5.87
Brick W130	142397391	142532205	6
Brick W131	142781620	143260581	6
Brick W132	144843115	145297023	6
Brick W133	145604826	145997393	6
Brick W134	147827089	147999739	4.25
Brick W135	148334579	148489680	4.4
Brick W136	149695320	150164447	4.92
Brick W137	151443415	151630522	5.96
Brick W138	151823100	151943226	3.78
Brick W139	152160343	152727248	6
Brick W140	153418765	153623166	3.13
Brick W141	154524261	154760604	5.99
Brick W142	154784324	155223020	6
Brick W143	155399282	155513175	3.14
Brick W144	156555873	157045222	5.83
Brick W145	157346573	157834279	5.94

Brick R114	93572970	94013217	5.94
Brick R115	94342482	94590085	5.97
Brick R116	94585670	94718353	-5.16
Brick R117	94891662	95053236	5.56
Brick R118	95623102	96429196	-6
Brick R119	100846739	101186148	4.13
Brick R120	101426971	101631761	6
Brick R121	101690475	101979240	6
Brick R122	102358675	102543938	-3.23
Brick R123	102574713	102731346	4.03
Brick R124	103238971	103359378	-3.01
Brick R125	103958732	104062180	4.85
Brick R126	104062187	104265219	-4.96
Brick R127	104520695	104652548	-4.65
Brick R128	105283381	105482393	6
Brick R129	106865089	107283301	-6
Brick R130	107283301	107461179	6
Brick R131	107524886	107700824	3.8
Brick R132	108055929	108153631	3.16
Brick R133	108901619	109487204	6
Brick R134	110833773	110946760	-5.84
Brick R135	112199953	112403352	3.41
Brick R136	112775983	113550158	6
Brick R137	113825303	114063500	6
Brick R138	114079872	114262124	6
Brick R139	114291544	114446308	-5.85
Brick R140	114434471	114638805	3.33
Brick R141	115080525	115372946	-5.84
Brick R142	115925271	116056108	-3.65
Brick R143	116056115	116137361	5.3
Brick R144	117218814	117398279	5.45
Brick R145	117445405	117670041	-6
Brick R146	117644636	117847476	5.82
Brick R147	117924292	118162063	-6
Brick R148	119978109	120109045	6
Brick R149	120554491	120743338	5.94
Brick R150	120789052	121017187	-6
Brick R151	121085955	121338474	-6
Brick R152	121626110	121788040	6
Brick R153	121897264	122387281	-6

Br114	127909488	128071521
Br115	128845851	129430706
Br116	130351158	130724696
Br117	130927537	131109951
Br118	131489429	131817836
Br119	132572685	132973060
Br120	135224329	135387963
Br121	135853182	136206649
Br122	136591783	136693312
Br123	137153317	137292571
Br124	137397736	137472952
Br125	137534304	137746516
Br126	138035969	138599071
Br127	139577057	139831092
Br128	140102624	140453222
Br129	141797513	141983177
Br130	142107055	142185005
Br131	142407482	142540122
Br132	143514503	143670892
Br133	143768445	144122271
Br134	144136153	144379728
Br135	144392132	144626683
Br136	145826435	145943756
Br137	146775909	146963091
Br138	147132173	147282013
Br139	147588364	147697511
Br140	147966507	148286101
Br141	149382259	149772968
Br142	150754037	150908683
Br143	151069294	151267015
Br144	152065741	152299832
Br145	152566499	153072971
Br146	153304879	153512805
Br147	153682296	153848098
Br148	154623004	154863273
Br149	156054513	156802085
Br150	156997666	157670156
Br151	158027006	158722877
Br152	158777768	159060763

Brick R154	123331138	123411195	-6
Brick R155	123443673	123563276	6
Brick R156	123862911	124060396	5.99
Brick R157	124222837	124513371	-6
Brick R158	125168371	125610171	6
Brick R159	126083472	126239101	-5.82
Brick R160	126239108	126398441	6
Brick R161	126382510	126545443	-6
Brick R162	127363482	127477380	-4.23
Brick R163	127969630	128603189	6
Brick R164	132029842	132157027	-6
Brick R165	132154758	132287521	5.96
Brick R166	133342990	133594575	-6
Brick R167	134563823	134884273	6
Brick R168	134924359	134986903	-4.18
Brick R169	134986910	135159799	5.76
Brick R170	135228370	135408791	6
Brick R171	135504951	135682503	4.36
Brick R172	135679443	135950641	-5.98
Brick R173	136165346	136455577	-3.79
Brick R174	136530935	136922519	-6
Brick R175	138661875	139178920	6
Brick R176	139432833	139530960	4.89
Brick R177	139529780	139648464	-3.89
Brick R178	140414849	140570277	-5.99
Brick R179	140667660	140946278	-6
Brick R180	141067463	141192687	6
Brick R181	141240664	141312985	-4.22
Brick R182	141535700	141732774	5.98
Brick R183	141827949	142041110	5.95
Brick R184	142127085	142276197	-6
Brick R185	142326197	142454193	-6
Brick R186	142510551	142623940	5.89
Brick R187	142850398	143212414	6
Brick R188	143305966	143347897	-6
Brick R189	143397897	143405293	-6
Brick R190	143417082	143990429	-6
Brick R191	144026258	144150460	-3.88
Brick R192	144345874	145239531	6
Brick R193	145216196	145470864	-4.91

Brick R194	145679599	146001215	6
Brick R195	146600443	146751929	-6
Brick R196	147652006	147793937	-5.8
Brick R197	147970671	148081425	3.78
Brick R198	148910537	149459433	-6
Brick R199	149575540	149958837	6
Brick R200	150307136	150528428	-6
Brick R201	151336593	151461322	3.22
Brick R202	151461322	151874387	-5.92
Brick R203	151878027	152078138	-4.93
Brick R204	152336116	152566493	4.27
Brick R205	152706057	152963177	-4.86
Brick R206	154479339	154655299	5.92
Brick R207	155035037	155252592	-5.97
Brick R208	155743039	155945601	4.5
Brick R209	156015302	156222348	-5.86
Brick R210	156326666	156645231	-6
Brick R211	156794116	156969452	3.85
Brick R212	157403923	157871489	6
Brick R213	157899930	158241077	4.95
Brick R214	158241084	158536928	-5.11

Value
6
6
5.88
3.79
6
6
6
5.67
3.47
5.99
5.27
5.77
6
6
6
3.04
5.93
6
6
5.07
4.52
6
5.94
4.18
4.07
3.08
4.99
5.98
5.93
6
3.3
3.43
5.13

WBS cells

	start coord	end coord	Value
Brick W1	397504	565182	3.7
Brick W2	607435	1474266	6
Brick W3	2608094	2853347	5.39
Brick W4	4593308	5052934	6
Brick W5	6737502	7076473	5.22
Brick W6	7357938	7638772	6
Brick W7	7989218	8222321	4.93
Brick W8	9304052	9524149	6
Brick W9	9798486	10009831	3.09
Brick W10	10668370	10780861	3.16
Brick W11	11022561	11205714	4.28
Brick W12	13768188	13927360	4.48
Brick W13	14110333	14331443	4.97
Brick W14	16309125	16623045	6
Brick W15	16803172	16962753	3.13
Brick W16	17299654	17475635	6
Brick W17	17552243	17702490	3.93
Brick W18	17715769	17907397	5.36
Brick W19	18137854	18232854	3.75
Brick W20	18515276	18659889	5.9
Brick W21	19411119	19544844	5.07
Brick W22	20545436	20884592	6
Brick W23	22538093	22751469	6
Brick W24	23382217	23586626	5.25
Brick W25	24658138	24818000	5.5
Brick W26	25406331	25581995	6
Brick W27	26084908	26205157	3.08
Brick W28	26240254	26695592	6
Brick W29	27382624	27507000	5.82
Brick W30	27626675	27798634	3.58
Brick W31	27819525	27985087	6
Brick W32	30217470	30319429	4.69
Brick W33	32675511	33073425	5.94

Ratio BRICKs

	start coord	end coord	Value
Brick R1	397504	619690	6
Brick R2	1105396	1539527	6
Brick R3	1766014	2022938	-5.84
Brick R4	2829127	3093143	-5.55
Brick R5	4149274	4751072	5.85
Brick R6	6188946	6630802	-5.98
Brick R7	6699472	7293493	6
Brick R8	7316279	8200338	6
Brick R9	8200338	8577131	-6
Brick R10	8968028	9042680	-6
Brick R11	9100172	9649641	6
Brick R12	9731291	10154886	6
Brick R13	10558057	10780861	6
Brick R14	10780867	10972660	-4.65
Brick R15	11180185	11453150	-6
Brick R16	13690806	14210776	6
Brick R17	14271771	14368832	6
Brick R18	14621207	14776390	-4.88
Brick R19	15087950	15258856	-4.16
Brick R20	15260446	15442050	6
Brick R21	16248809	16624946	6
Brick R22	16818889	16986313	6
Brick R23	17552243	17702490	4.5
Brick R24	17862462	18002075	5.37
Brick R25	18137854	18243058	4.02
Brick R26	18409051	18708936	6
Brick R27	19293584	19982444	6
Brick R28	20707263	20894209	5.8
Brick R29	21785504	22180700	-5.77
Brick R30	22244778	22591586	-6
Brick R31	24459477	24637735	-5.92
Brick R32	25114730	25273936	-5.3
Brick R33	25508309	25624719	-5.96

6	Brick W34	33469177	33603541	4.34	Brick R34	25954582	26141971	5.77
6	Brick W35	34038919	34161990	5.03	Brick R35	26247948	26694206	6
6	Brick W36	34919184	35075418	5.73	Brick R36	27499057	27547916	-4.43
4.85	Brick W37	36124430	36366208	6	Brick R37	27547916	28020854	6
5.97	Brick W38	38088344	38569284	6	Brick R38	28546982	28671417	-6
6	Brick W39	39364973	39468287	5.49	Brick R39	28744269	28862195	5.43
6	Brick W40	40371807	40528568	3.43	Brick R40	28862201	29010186	-5.89
6	Brick W41	43373730	43636735	5.89	Brick R41	29241138	29386140	-6
5.97	Brick W42	43763639	44000010	6	Brick R42	30217470	30319429	3.87
4.51	Brick W43	44037523	44394834	6	Brick R43	31697030	31918277	-5.67
6	Brick W44	44461794	44704376	6	Brick R44	33348720	33599508	5.9
5.79	Brick W45	45179918	45545146	4.6	Brick R45	33652573	33971071	-6
6	Brick W46	47040848	47215520	6	Brick R46	34150276	34363052	5.14
6	Brick W47	47223524	47590269	6	Brick R47	36118814	36626148	6
3.07	Brick W48	47987036	48401308	5.96	Brick R48	37069475	37382826	-5.99
2.01	Brick W49	51411254	51679331	3.54	Brick R49	37388388	37517298	5.79
4.45	Brick W50	52394547	52655694	6	Brick R50	38029022	38225523	-5.16
1.32	Brick W51	53411638	53534571	1.87	Brick R51	38225544	38584484	6
2.63	Brick W52	54187445	54405947	1.08	Brick R52	38813780	39030533	-4.82
5.7	Brick W53	54688032	54983090	6	Brick R53	39366293	39469556	6
5.23	Brick W54	55059424	55209739	3.84	Brick R54	40188890	40319339	-4.12
2.4	Brick W55	55431321	56003355	6	Brick R55	40318751	40478015	6
1.18	Brick W56	56247757	56493108	1.09	Brick R56	40506411	40637182	6
1.11	Brick W57	56695240	57427538	6	Brick R57	40914410	41256066	-6
3.73	Brick W58	57930662	58054331	6	Brick R58	43535231	43701728	-3.74
2.46	Brick W59	61054331	61310513	6	Brick R59	44461794	44704376	3.19
3.25	Brick W60	61360513	61460465	6	Brick R60	47004752	47172598	4.77
5.22	Brick W61	61510465	61677020	6	Brick R61	48259766	48458678	-5.61
6	Brick W62	61727020	61917157	6	Brick R62	50599476	50786331	-3.28
1.19	Brick W63	61967157	62830528	6	Brick R63	51652081	52037189	-5.83
5.15	Brick W64	63596674	63887897	2.53	Brick R64	52361151	52775134	6
4.85	Brick W65	64592649	64879006	1.58	Brick R65	53363658	53474034	6
1.12	Brick W66	64948440	65179097	3.61	Brick R66	53487593	53764390	6
1.07	Brick W67	66165187	66335724	1.1	Brick R67	54178195	54438636	4.71
3.77	Brick W68	69490876	69625329	1.05	Brick R68	54783659	54830134	1.07
4.47	Brick W69	70171660	70325127	1.28	Brick R69	54936952	55067588	-1.51
3.09	Brick W70	70832606	71001236	2.8	Brick R70	55913173	56252009	-4.55
4.12	Brick W71	72084069	72420645	2.71	Brick R71	56824394	57087488	-1.66
6	Brick W72	75100386	75432250	6	Brick R72	57326726	57489660	-1.3
6	Brick W73	75852355	76327818	6	Brick R73	57842145	58054331	1.2

6	Brick W74	76451835	76672776	5.85	Brick R74	61054331	61310513	1.2
5.97	Brick W75	77459889	77641208	1.95	Brick R75	61360513	61460465	1.2
5.13	Brick W76	78138793	78341901	6	Brick R76	61510465	61677020	1.2
5.93	Brick W77	78559792	78720335	3.92	Brick R77	61727020	61917157	1.2
5.42	Brick W78	80667397	80955831	5.25	Brick R78	61967157	62501121	1.2
3.99	Brick W79	82612881	82779953	3.22	Brick R79	63019394	63247917	-1.72
6	Brick W80	83021332	83210247	6	Brick R80	65353463	65635417	-1
5.93	Brick W81	85517790	85750622	5.84	Brick R81	66422895	66539510	-1.09
5.82	Brick W82	85952526	86117505	3.41	Brick R82	67614036	67821154	-2.29
5.99	Brick W83	86518338	86683154	5.82	Brick R83	67856289	68007351	-3.48
5.95	Brick W84	87071657	87325045	5.11	Brick R84	68792511	69044297	-1.71
5.18	Brick W85	88965135	89123466	5.94	Brick R85	69654207	69817575	-1.51
6	Brick W86	89866208	90046990	3.69	Brick R86	70034766	70160458	-2.18
6	Brick W87	90137234	90313997	3.81	Brick R87	70579145	70834443	-4.66
3.47	Brick W88	90682631	90783699	3.77	Brick R88	73011191	73323144	-4.11
6	Brick W89	92398259	92504885	6	Brick R89	75085813	75295314	1.06
6	Brick W90	93619454	93804364	5.76	Brick R90	75295314	75782940	-6
4.52	Brick W91	95020803	95392623	6	Brick R91	75854598	75999340	-1.49
6	Brick W92	96730919	97095234	6	Brick R92	76009860	76155020	1.24
3.36	Brick W93	97541016	97963491	5.65	Brick R93	76342606	76480218	-3.41
3.21	Brick W94	98188893	98407820	5.94	Brick R94	76562193	76644853	1.14
3.56	Brick W95	98595179	98814969	6	Brick R95	76648977	76923064	-5.9
4.61	Brick W96	100019738	100434558	6	Brick R96	77052335	77186773	-3.44
6	Brick W97	101691047	102441354	6	Brick R97	77858111	78082480	-1.24
5.97	Brick W98	104081510	104261571	5.81	Brick R98	78138793	78318097	4.68
5.85	Brick W99	104767608	105347843	5.81	Brick R99	78318103	78478469	-5.27
5.84	Brick W100	106803722	107050631	3.36	Brick R100	79709344	80023357	-3.76
6	Brick W101	107328480	107461179	3.94	Brick R101	80456089	81043107	6
3.05	Brick W102	107883260	108070354	3.72	Brick R102	81043107	81301253	-5.83
5.18	Brick W103	108132772	108468530	5.9	Brick R103	83941850	84095983	-3.34
3.75	Brick W104	109181931	109428911	4.67	Brick R104	84193243	84484849	6
6	Brick W105	110327687	110664094	6	Brick R105	84514595	84691909	-6
6	Brick W106	110872566	111289491	6	Brick R106	85401518	85874171	5.7
3.35	Brick W107	111645991	111961700	5.85	Brick R107	86411037	86591450	-3.25
3.06	Brick W108	113228608	113486850	4.61	Brick R108	87242287	87476110	-4.59
6	Brick W109	113560193	113782494	5.97	Brick R109	87623078	87776101	-5.63
6	Brick W110	113818351	114056611	4.28	Brick R110	88036123	88246219	-6
3.69	Brick W111	114508476	114688496	6	Brick R111	88342970	88555553	-4.88
6	Brick W112	116305231	116624288	5.96	Brick R112	88847415	89332332	6
3.61	Brick W113	117847475	118002822	6	Brick R113	89682713	89804313	-3.49

5.95	Brick W114	119655766	119859287	4.85	Brick R114	89906905	90075683	-4.19
6	Brick W115	120129589	120473539	6	Brick R115	90173771	90339628	6
6	Brick W116	121560089	121966236	6	Brick R116	90384043	90555168	-5.21
6	Brick W117	122702568	122903646	6	Brick R117	90648694	90765050	6
6	Brick W118	122930374	123193429	6	Brick R118	90768204	90914381	-6
6	Brick W119	123870007	124094698	6	Brick R119	91043860	91234291	-4.69
6	Brick W120	125040289	125232743	5.29	Brick R120	91296775	91528356	-5.34
5.64	Brick W121	126052649	126159935	6	Brick R121	92252702	92418581	3.03
4.42	Brick W122	126362509	126678547	6	Brick R122	92419249	92576744	-4.43
4.16	Brick W123	130351158	130584437	5.88	Brick R123	92770282	93110360	-6
4.51	Brick W124	133724697	133859580	5.82	Brick R124	94293175	94585664	-3.92
5.99	Brick W125	134077151	134427271	6	Brick R125	94603791	94732900	4.19
6	Brick W126	135548957	135701591	4.08	Brick R126	94812077	95174672	-5.87
5.95	Brick W127	136582072	137000346	6	Brick R127	95325845	95503975	-3.68
6	Brick W128	137437031	137586609	5.99	Brick R128	95959485	96178764	-6
4.11	Brick W129	137760780	138086034	5.81	Brick R129	98081466	98254744	6
3.17	Brick W130	138190731	138626086	6	Brick R130	98314628	98520386	-4.09
3.77	Brick W131	139279861	139379377	6	Brick R131	98520392	98858825	5.96
3.58	Brick W132	139404377	139471113	6	Brick R132	100165833	100332404	4.03
6	Brick W133	139587008	139760462	5.98	Brick R133	101343540	101610686	-5.51
6	Brick W134	140045143	140440045	6	Brick R134	101923208	102214038	3.04
6	Brick W135	140862892	140992429	5.28	Brick R135	102485949	102723466	-5.54
3.27	Brick W136	140996632	141058617	5.57	Brick R136	103170590	103306380	-5.34
5.92	Brick W137	141461230	141631058	5.73	Brick R137	104188409	104449722	-6
3.89	Brick W138	142461775	142964573	6	Brick R138	104610187	104858487	-3.2
5.99	Brick W139	143912886	144064119	4.99	Brick R139	105035575	105441828	-6
5.99	Brick W140	144153301	144411547	5.09	Brick R140	106698986	106926010	6
5.68	Brick W141	145315810	145499716	5.81	Brick R141	107328480	107501886	6
3.38	Brick W142	147827609	148039810	6	Brick R142	107642640	107828809	4.71
5.99	Brick W143	148426029	149336309	6	Brick R143	107835671	107982300	-3.66
6	Brick W144	149804007	150023046	5.53	Brick R144	107990068	108196511	-6
6	Brick W145	150326221	150557825	6	Brick R145	108262345	108423404	3.41
5.46	Brick W146	151103608	151285443	5.99	Brick R146	109124961	109223601	3.92
5.94	Brick W147	151385518	151842541	5.77	Brick R147	109372281	109892759	-6
6	Brick W148	151897127	152101494	6	Brick R148	110240927	110644634	5.91
6	Brick W149	152687644	152951737	5.29	Brick R149	110870129	111289491	6
6	Brick W150	154102332	154270634	3.19	Brick R150	111708148	112016863	5.78
6	Brick W151	154370634	154428316	3.19	Brick R151	112844537	113200789	-6
3.53	Brick W152	154695209	155094900	6	Brick R152	113345870	113520217	5.89
	Brick W153	155162695	155536308	5.98	Brick R153	113531609	113739897	6

Brick W154	155781550	156410627	6
Brick W155	156568088	157196100	6
Brick W156	157376934	158707091	6
Brick W157	158732703	158971224	6

Brick R154	113825302	114056611	3.13
Brick R155	114570059	114696586	5.96
Brick R156	114794557	114941253	-3.21
Brick R157	115977726	116165039	-6
Brick R158	116305231	116624288	5.71
Brick R159	119270565	119688576	6
Brick R160	119691605	119931709	-6
Brick R161	120193229	120311702	6
Brick R162	121517092	121838838	6
Brick R163	122622906	122975715	6
Brick R164	123459072	123828963	-6
Brick R165	123924075	124180280	6
Brick R166	124963937	125335672	5.8
Brick R167	126070964	126145915	5.03
Brick R168	127811597	128265351	-5.97
Brick R169	128789436	129416469	-6
Brick R170	130254523	130370486	-5.91
Brick R171	130516566	130719618	-6
Brick R172	130918644	131477479	-6
Brick R173	131481023	131916909	-6
Brick R174	132541174	132903881	-5.87
Brick R175	133684578	133884894	6
Brick R176	133982840	134406718	6
Brick R177	135370738	135520329	-6
Brick R178	135689258	135868097	3.99
Brick R179	135868103	136111052	-4.64
Brick R180	136301685	136591777	5.83
Brick R181	136612317	136986598	5.88
Brick R182	137278654	137398990	-4.53
Brick R183	137430560	137608607	6
Brick R184	137591518	137763994	-6
Brick R185	137761446	138040534	3.73
Brick R186	138029465	138256657	-6
Brick R187	138321576	138492674	-3.07
Brick R188	139178926	139354178	3.82
Brick R189	139546125	139660964	3.18
Brick R190	139677171	139831092	-3.93
Brick R191	140166100	140246735	4.42
Brick R192	141404738	141593134	6
Brick R193	141672929	142048195	-6
