# Supplementary material for: Structural Variation-Associated Expression Changes Are Paralleled by Chromatin Architecture Modifications
Source: PLoS One. 2013 Nov 12;8(11):e79973. doi: 10.1371/journal.pone.0079973 (PMC3827143; doi:10.1371/journal.pone.0079973)
Supplement: Table S3 — Correlation analyses between six different marks of regulatory elements and interacting regions in Ctrl cells (Ctrl Bricks), in all differential interacting regions significantly decreased (negative ratio Bricks) or increased (positive ratio Bricks) in WBS versus Ctrl cells. Permutation test with number of permutation = 1000. Significant p-values are highlighted in grey. (PDF) [file pone.0079973.s010.pdf]

Supplementary Table S3. Correlation analyses between six different marks of regulory elements and interacting regions in Ctrl cells (Ctrl Bricks), in all differential interacting regions (Ratio Bricks), in interacting regions significantly decreased (negative ratio Bricks) or increased (positive ratio Bricks) in WBS versus Ctrl cells. Permutation test with number of permutations=1000. Significant p-values are highlighted in grey.

		**Ctrl Bricks**							**Ratio Bricks**							**negative ratio Bricks**							**positive ratio Bricks**						
Mark	viewpoints	GBAS	ZNF107	ASL	KCTD7	HIP1	POR	MDH2	GBAS	ZNF	ASL	KCTD7	HIP1	POR	MDH2	GBAS	ZNF	ASL	KCTD7	HIP1	POR	MDH2	GBAS	ZNF	ASL	KCTD7	HIP1	POR	MDH2
CTCF	number of peaks overlapping Bricks	832	917	1181	1183	1262	1204	1102	1001	1087	733	978	1130	919	348	470	627	335	499	664	474	180	531	460	398	479	466	445	168
	% of total peaks	27	30	39	39	42	40	36	33	36	24	32	37	30	11	15	21	11	16	22	16	6	17	15	13	16	15	15	6
	p-value	0.032	0.017	0.001	0.001	0.001	0.001	0.001	1.000	0.001	0.028	1.000	0.001	0.001	1.000	0.998	0.001	0.271	0.999	0.001	0.001	1.000	0.969	0.001	0.027	0.999	0.014	0.001	1.000
DNaseI	number of peaks overlapping Bricks	2747	2881	4013	4211	4391	4559	4211	2898	3021	2017	2761	3483	2766	940	1236	1715	883	1555	2011	1574	580	1662	1306	1134	1206	1472	1192	360
	% of total peaks	31	32	45	47	49	51	47	33	34	23	31	39	31	11	14	19	10	18	23	18	7	19	15	13	14	17	13	4
	p-value	0.004	0.01	0.001	0.001	0.001	0.001	0.001	0.001	0.001	0.001	0.001	0.001	0.001	0.694	0.001	0.001	0.001	0.001	0.001	0.001	0.434	0.001	0.001	0.001	0.001	0.001	0.001	0.822
FAIRE	number of peaks overlapping Bricks	2103	2184	2600	2824	2673	2672	2456	2452	2500	1638	2202	2519	2005	836	1099	1472	734	1167	1443	1075	485	1353	1028	904	1035	1076	930	351
	% of total peaks	30	31	37	40	38	38	35	34	35	23	31	35	28	12	15	21	10	16	20	15	7	19	14	13	15	15	13	5
	p-value	0.001	0.003	0.001	0.001	0.001	0.001	0.001	0.021	0.001	0.001	0.407	0.001	0.001	1.000	0.418	0.001	0.001	0.274	0.001	0.001	1.000	0.005	0.001	0.001	0.545	0.001	0.001	1.000
H3K27ac	number of peaks overlapping Bricks	964	991	1372	1494	1524	1539	1437	915	1069	610	982	1162	928	307	405	585	255	506	656	563	210	510	484	355	476	506	365	97
	% of total peaks	32	33	46	50	51	51	48	31	36	20	33	39	31	10	14	20	9	17	22	19	7	17	16	12	16	17	12	3
	p-value	0.004	0.019	0.001	0.001	0.001	0.001	0.001	1.000	0.001	1.000	1.000	0.025	0.001	1.000	1.000	0.001	1.000	1.000	0.094	0.001	1.000	1.000	0.001	0.873	1.000	0.061	0.014	1.000
H3K4me	number of peaks overlapping Bricks	2126	2219	2988	3041	3052	3185	3019	2328	2545	1690	2154	2556	2226	844	1038	1444	799	1152	1497	1282	565	1290	1101	891	1002	1059	944	279
	% of total peaks	31	32	43	44	44	46	44	34	37	24	31	37	32	12	15	21	12	17	22	19	8	19	16	13	14	15	14	4
	p-value	0.003	0.009	0.001	0.001	0.001	0.001	0.001	0.618	0.001	0.001	0.978	0.001	0.001	1.000	0.738	0.001	0.001	0.427	0.001	0.001	1.000	0.362	0.001	0.001	0.998	0.001	0.001	1.000
p300	number of peaks overlapping Bricks	50	54	77	88	87	91	87	51	41	39	47	64	48	14	22	20	13	33	32	23	9	29	21	26	14	32	25	5
	% of total peaks	32	35	50	57	56	59	56	33	27	25	31	42	31	9	14	13	8	21	21	15	6	19	14	17	9	21	16	3
	p-value	0.089	0.093	0.001	0.001	0.001	0.001	0.001	1	0.932	1	1	1	1	1	1	0.895	1	1	1	1	1	1	0.798	1	1	1	1	1
